# YAP Activity Is Necessary and Sufficient for Basal Progenitor Abundance and Proliferation in the Developing Neocortex

**DOI:** 10.1016/j.celrep.2019.03.091

**Published:** 2019-04-23

**Authors:** Milos Kostic, Judith T.M.L. Paridaen, Katherine R. Long, Nereo Kalebic, Barbara Langen, Nannette Grübling, Pauline Wimberger, Hiroshi Kawasaki, Takashi Namba, Wieland B. Huttner

**Affiliations:** 1 Max Planck Institute of Molecular Cell Biology and Genetics, Pfotenhauerstrasse 108, 01307 Dresden, Germany; 2 Technische Universität Dresden, Universitätsklinikum Carl Gustav Carus, Klinik und Poliklinik für Frauenheilkunde und Geburtshilfe, Fetscherstraße 74, 01307 Dresden, Germany; 3 Department of Medical Neuroscience, Graduate School of Medical Sciences, Kanazawa University, Ishikawa 920-8640, Japan

**Keywords:** Hippo signaling, YAP activity, neocortical evolution, SVZ, basal progenitors

## Abstract

Neocortex expansion during mammalian evolution has been linked to an increase in proliferation of basal progenitors in the subventricular zone. Here, we explored a potential role of YAP, the major downstream effector of the Hippo pathway, in proliferation of basal progenitors. YAP expression and activity are high in ferret and human basal progenitors, which exhibit high proliferative capacity, but low in mouse basal progenitors, which lack such capacity. Conditional expression of a constitutively active YAP in mouse basal progenitors resulted in increased proliferation of basal progenitor and promoted production of upper-layer neurons. Pharmacological and genetic interference with YAP function in ferret and human developing neocortex resulted in decreased abundance of cycling basal progenitors. Together, our data indicate that YAP is necessary and sufficient to promote the proliferation of basal progenitors and suggest that increases in YAP levels and presumably activity contributed to the evolutionary expansion of the neocortex.

## Highlights

• Higher YAP levels in ferret and human than in mouse basal progenitors of fetal neocortex
• Increasing YAP activity in mouse basal progenitors (BPs) is sufficient to expand them
• YAP is required to maintain the abundance of BPs in ferret and human fetal neocortex

## Graphical Abstract

**Figure:**
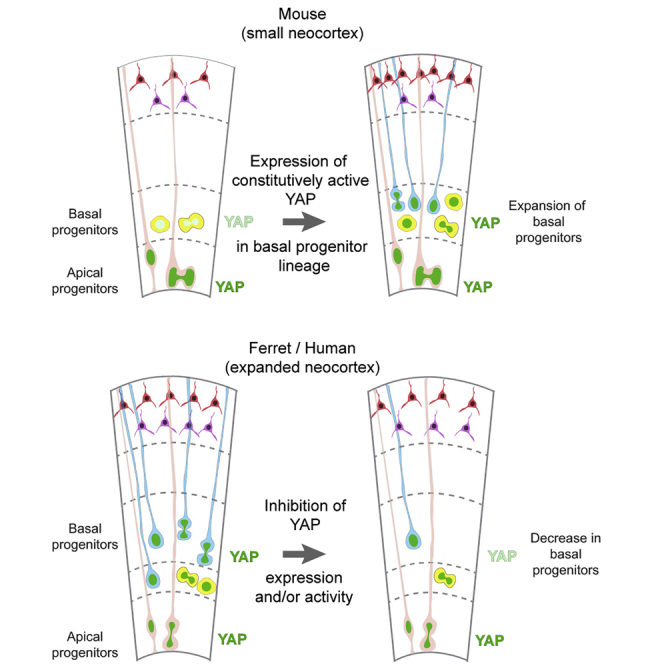

## Teaser

Kostic et al. demonstrate that YAP expression and activity in developing neocortex are higher in ferret and human than mouse and are required and sufficient for an abundance of basal progenitors. This suggests that increases in YAP expression and activity levels contributed to the evolutionary expansion of the neocortex.

## Introduction

The neocortex, the seat of higher cognitive functions, undergoes substantial expansion during the evolution of certain mammalian brains such as human. A major factor in neocortical expansion, notably regarding the increase in the number of cortical neurons, is thought to be an increased proliferative capacity of cortical neural progenitor cells (cNPCs) (Fietz and Huttner, 2011, Florio and Huttner, 2014, Geschwind and Rakic, 2013, Lui et al., 2011, Namba and Huttner, 2017, Rakic, 2009, Wilsch-Bräuninger et al., 2016).

Two principal classes of cNPCs exist in the developing neocortex, referred to as apical progenitors (APs) and basal progenitors (BPs) (Florio and Huttner, 2014, Lui et al., 2011, Namba and Huttner, 2017). The defining feature of APs is that they undergo mitosis at the ventricular (apical) surface of the ventricular zone (VZ), the primary germinal zone where the AP cell bodies reside (Florio and Huttner, 2014, Namba and Huttner, 2017). At the onset of neurogenesis, apical (or ventricular) radial glia (aRG) are the major AP cell type (Fietz and Huttner, 2011, Florio and Huttner, 2014, Lui et al., 2011, Namba and Huttner, 2017). The defining feature of BPs is that they undergo mitosis away from the apical surface, typically in a secondary germinal zone called the subventricular zone (SVZ) where the BP cell bodies reside (Haubensak et al., 2004, Miyata et al., 2004, Noctor et al., 2004). BPs originate from APs, delaminate from the apical surface, migrate beyond the VZ, and thus form the SVZ. There are two main types of BPs, basal intermediate progenitors (bIPs) and basal (or outer) radial glia (bRG). In contrast to aRG, which are epithelial cells exhibiting apical-basal polarity with contact to the ventricle and (in the canonical form) to the basal lamina, bIPs are non-epithelial cells that no longer exhibit apical-basal polarity and have lost contact with both the ventricle and the basal lamina (Haubensak et al., 2004, Miyata et al., 2004, Noctor et al., 2004). bRG, however, though lacking an apical process that reaches the ventricle, retain epithelial features in that they (in the canonical form) possess a basal process that contacts the basal lamina (Betizeau et al., 2013, Fietz et al., 2010, Hansen et al., 2010, Reillo et al., 2011).

BP composition and proliferative capacity may differ greatly between a developing lissencephalic neocortex (e.g., mouse) and a developing gyrencephalic neocortex (e.g., ferret and human). In the developing mouse neocortex, BPs mostly comprise bIPs that typically undergo neurogenic consumptive divisions giving rise to two neurons; compared to the aRG they derive from, these neurogenic bIPs characteristically upregulate the transcription factor Tbr2 and downregulate the transcription factor Sox2 (Haubensak et al., 2004, Miyata et al., 2004, Noctor et al., 2004). Only a minor portion of mouse BPs are bRG, and their proliferative potential is limited (Shitamukai et al., 2011, Wang et al., 2011). In contrast, in the developing ferret and human neocortex, the majority of BPs are proliferative bRG (highly proliferative in human) that do not express Tbr2 but rather maintain expression of Sox2 (Fietz et al., 2010, Hansen et al., 2010, Reillo et al., 2011). Moreover, as first shown in a seminal contribution for the developing monkey neocortex (Smart et al., 2002), the SVZ in a gyrencephalic neocortex is characteristically split into two morphologically distinct zones, an inner SVZ (iSVZ) and an outer SVZ (oSVZ). Of note, the evolutionary expansion of the neocortex has been linked to an increase in the proliferative capacity and abundance of BPs in the oSVZ, especially of bRG (Dehay et al., 2015, Fietz and Huttner, 2011, Lui et al., 2011, Namba and Huttner, 2017). However, the molecular players that differentially promote the proliferative capacity of BPs across the various mammalian species remain largely unknown.

To gain insight into this issue, we examined a major molecular mechanism known to regulate organ size, the Hippo-YAP signaling pathway (Barry and Camargo, 2013, Camargo et al., 2007, Lian et al., 2010, Yu et al., 2015). The core of Hippo-YAP signaling is the YAP protein, whose ability to activate transcription is regulated by phosphorylation. Phosphorylated YAP (phospho-YAP) is largely retained in the cytoplasm, whereas dephosphorylated YAP (dephospho-YAP) can translocate to the nucleus and activate the expression of genes linked to cell proliferation (Zanconato et al., 2015, Zhao et al., 2007, Zhao et al., 2008, Zhao et al., 2010). Recent studies dissecting the roles of the cadherin family members Dchs1 and Fat4 (Cappello et al., 2013) and the tumor suppressor neurofibromatosis 2 (Lavado et al., 2013, Lavado et al., 2014) and investigating heterotopia formation (Saito et al., 2018) in mouse brain development have reported that YAP promotes the proliferation of mouse APs. These studies, however, have not focused on a potential role of YAP in regulating the proliferation of BPs, nor have they addressed whether differences in YAP activity may underlie the differences in the proliferative capacity of BPs across various mammalian species in the context of the evolutionary expansion of the neocortex.

In the present study, we have identified differences in YAP expression and YAP activity between the developing lissencephalic mouse and gyrencephalic ferret and human neocortex that match the differences in the proliferative capacity of BPs across these species. Enhancing YAP activity in mouse BPs induced their proliferation and therefore shifted their fate from neurogenic to proliferative. In contrast, inhibition of endogenous YAP activity by verteporfin, administration of a dominant-negative YAP, or CRISPR-Cas9-mediated disruption of YAP expression reduced BP proliferation in developing ferret and human neocortex. Taken together, these findings suggest that an upregulation of YAP levels and presumably activity contributed to the increased proliferative capacity of BPs in the context of the evolutionary expansion of the neocortex.

## Results

### BPs with High Proliferative Capacity, which Are Abundant in Embryonic Ferret and Fetal Human Neocortex but Lacking in Embryonic Mouse Neocortex, Show High YAP Expression

We first exploited previously published transcriptome datasets (Fietz et al., 2012, Florio et al., 2015) to analyze the levels of *Yap*-*YAP* mRNA in the germinal zones and cNPC classes of developing mouse and human neocortex (Figure S1). *Yap*-*YAP* mRNA was robustly expressed in the VZ of both embryonic day 14.5 (E14.5) mouse and 13 weeks post-conception (13 wpc) human neocortex (Figure S1A) and accordingly in mouse and human aRG (Figure S1B). Moreover, *Yap* expression was 2-fold higher in *Tis21*-GFP-negative (i.e., proliferative) aRG than *Tis21*-GFP-positive (i.e., BP-genic) aRG of E14.5 mouse neocortex (Figure S1C; see below for the specificity of *Tis21* gene expression) (Florio et al., 2015). Strikingly, *Yap*-*YAP* mRNA was found to be expressed in the human iSVZ and oSVZ, but not the mouse SVZ (Figure S1A), and in human bRG, but not mouse BPs (Figure S1B). Given that both human and mouse proliferative APs and human, but not mouse, BPs are endowed with the ability to expand their population size by cell proliferation (Namba and Huttner, 2017), these data provided a first indication that the proliferative capacity of cNPCs, notably of BPs, may be linked to the expression of YAP. Consistent with this notion, no significant *Yap*-*YAP* mRNA expression was detected in the mouse and human cortical plate (CP) (Figure S1A) or in post-mitotic neurons (Figure S1B).

Comparison of mRNA levels between a prospective gyrus versus a prospective sulcus of developing (postnatal day 2 [P2]) ferret neocortex, available in a previously published transcriptome dataset (de Juan Romero et al., 2015), showed that the *Yap* mRNA level was higher in the oSVZ of the prospective gyrus than the prospective sulcus (Figure S1D), consistent with the notion that a relative increase in cNPC proliferation in this germinal zone contributes to gyrus formation (Hansen et al., 2010, Reillo et al., 2011, Wang et al., 2011). Taken together, these *Yap*-*YAP* mRNA data raised the possibility not only that YAP may have a role in the proliferation of APs, as previously shown for embryonic mouse neocortex (Lavado et al., 2013, Lavado et al., 2014), but also that differences in the level of active YAP may underlie the differences in the proliferative capacity of mouse versus ferret and human BPs.

**Figure 1:**
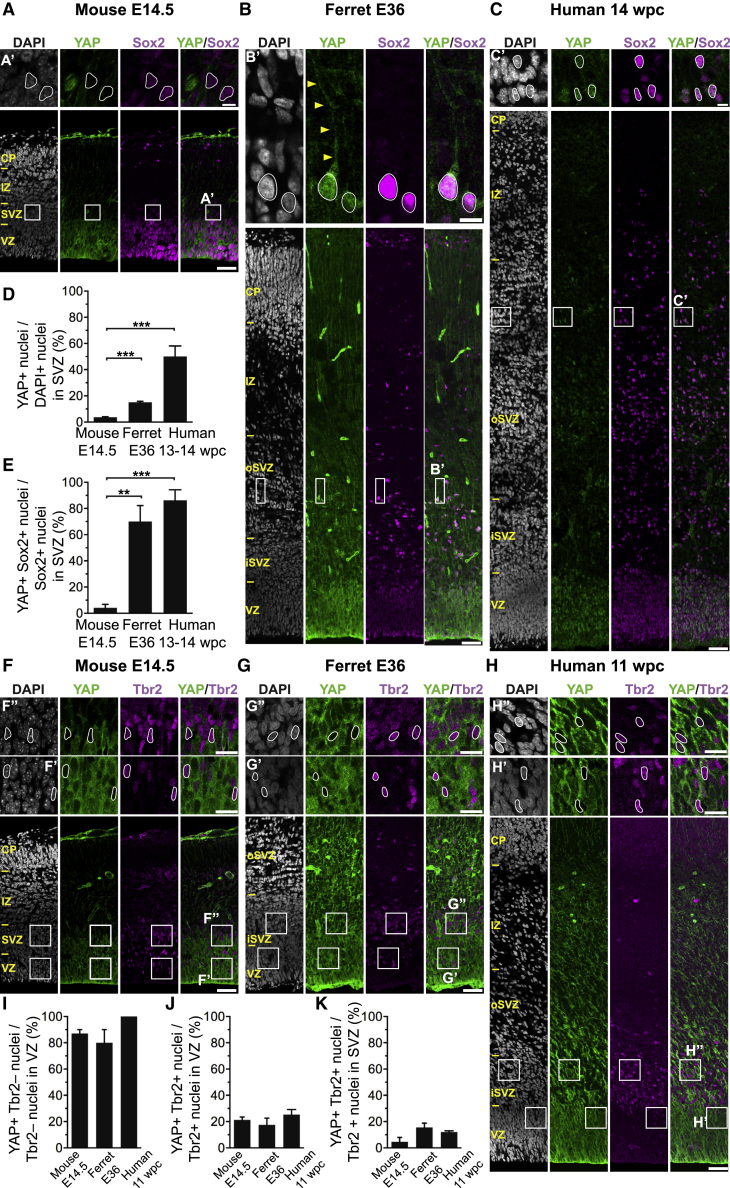
The Majority of Ferret and Human, but Not Mouse, Sox2-Positive Tbr2-Negative BPs Exhibit Nuclear YAP (A–C) Double immunofluorescence for YAP (green) and Sox2 (magenta), combined with DAPI staining (white), of mouse E14.5 (A), ferret E36 (B), and human 14 wpc (C) neocortex. Boxes indicate areas in the SVZ (A) and oSVZ (B and C) that are shown at higher magnification (A′, B′, and C′); selected Sox2-positive nuclei that are YAP negative in mouse and YAP positive in ferret and human are outlined by white lines; arrowheads indicate a YAP-positive basal process of a bRG. (D and E) Quantification of the percentage of DAPI-stained nuclei (D) and Sox2-positive nuclei (E) in the SVZ that are YAP positive in mouse E14.5, ferret E36, and human 13–14 wpc neocortex. Two or three images per embryo-fetus were taken, 30 randomly picked DAPI-stained nuclei (D) and Sox2-positive nuclei (E) in the SVZ were scored per image, and the values obtained were averaged for each embryo-fetus. Data are the mean of four embryos-fetuses. (F–H) Double immunofluorescence for YAP (green) and Tbr2 (magenta), combined with DAPI staining (white), of mouse E14.5 (F), ferret E36 (G), and human 11 wpc (H) neocortex. Boxes indicate areas in the VZ and SVZ (F) or iSVZ (G and H) that are shown at higher magnification (F′, F″, G′, G″, H′, and H″), as indicated; selected Tbr2-positive nuclei that are YAP negative in mouse, ferret, and human are outlined by white lines. (I–K) Quantification of the percentage of Tbr2-negative nuclei in the VZ (I), Tbr2-positive nuclei in the VZ (J), and Tbr2-positive nuclei in the SVZ (K) that are YAP positive in mouse E14.5, ferret E36, and human 11 wpc neocortex. Two or three images per embryo-fetus were taken, 30 randomly picked Tbr2-negative nuclei in the VZ (I) and Tbr2-positive nuclei in the VZ (J) and SVZ (K) were scored per image, and the values obtained were averaged for each embryo-fetus. Data are the mean of three or four embryos-fetuses. (A–C and F–H) Images are 1-μm optical sections. Scale bars represent 50 μm (A–C and F–H), 10 μm (A′, B′, and C′), and 20 μm (F′, F″, G′, G″, H′, and H″). (D, E, and I–K) Error bars indicate SEM; ^∗∗^p < 0.01, ^∗∗∗^p < 0.001 (one-way ANOVA, post hoc Tukey HSD). See also Figures S1 and S2.

We therefore examined the expression of the YAP protein in embryonic mouse, embryonic ferret, and fetal human neocortex by immunofluorescence (Figures 1A–1C and 1F–1H). Consistent with the mRNA expression data (Figure S1A), YAP immunoreactivity was overt in the E14.5 mouse, E36 ferret, and 14 wpc human VZ and in the ferret and human SVZ, notably the oSVZ, but was low in the mouse SVZ (Figures 1A–1C). In the case of the embryonic ferret oSVZ, YAP immunostaining revealed cells exhibiting a basal process (Figure 1B′), suggesting that they were bRG.

### YAP-Expressing BPs in Embryonic Ferret and Fetal Human Neocortex Are Sox2 Positive

For YAP to be able to promote the expression of genes linked to proliferation, it needs to be nuclear (Zanconato et al., 2015, Zhao et al., 2007, Zhao et al., 2008, Zhao et al., 2010). Interestingly, in line with a potential role of YAP in BP proliferation, the 13–14 wpc human SVZ contained the highest percentage of YAP-positive nuclei (50%), followed by the E36 ferret SVZ (15%), whereas the E14.5 mouse SVZ exhibited low levels of YAP-positive nuclei (4%) (Figure 1D).

We sought to obtain direct evidence that the higher level of nuclear YAP protein seen in embryonic ferret and fetal human SVZ as compared to mouse SVZ (Figure 1D) is linked to the increased proliferative capacity of ferret and human BPs versus mouse BPs. To this end, we compared the expression of YAP with that of Sox2 (Figures 1A–1C), an indicator of proliferative capacity (Hansen et al., 2010, Reillo et al., 2011, Wang et al., 2011). Specifically, we asked whether SVZ nuclei positive for Sox2 are also positive for YAP. Indeed, the vast majority of the Sox2-positive BP nuclei in the E36 ferret and 13–14 wpc human SVZ were YAP positive (70% and 86%, respectively), whereas this was the case for only a minor proportion of the Sox2-positive nuclei in the E14.5 mouse SVZ (4%) (Figure 1E).

### YAP in Sox2-Positive BP Nuclei in Embryonic Ferret and Fetal Human Neocortex, in Contrast to that in Embryonic Mouse Neocortex, Is Mostly Dephosphorylated

Phosphorylation of YAP at specific serine residues (serine 127 in the case of human YAP) leads to its interaction with 14-3-3 proteins, which in turn results in the retention of YAP in the cytoplasm, rendering it unable to activate transcription (Moya and Halder, 2019). However, phospho-YAP is not necessarily completely retained in the cytoplasm but can also in part be localized in the nucleus (Das et al., 2016). Within the nucleus, YAP phosphorylated at serine 127 tends to interact more with p73, resulting in activation of genes involved in apoptosis, rather than with TEA domain transcription factor (TEAD), which would result in activation of genes involved in cell proliferation (Downward and Basu, 2008, Matallanas et al., 2007). We therefore investigated whether the YAP protein in Sox2-positive BP nuclei was in a dephosphorylated state. At first, qualitative indication that this was the case was obtained by comparing the immunofluorescence signals for total YAP and phospho-YAP in Sox2-positive BP nuclei in the SVZ of human 11 wpc neocortex (Figure S2A). Relative to the cytoplasmic signals for total YAP and phospho-YAP, respectively, this comparison showed a much lower signal for nuclear phospho-YAP than nuclear total YAP, suggesting that most nuclear YAP in fetal human neocortical BPs was in the dephosphorylated state. We therefore used this approach to compare the relative levels of dephospho-YAP in Sox2-positive BP nuclei in the SVZ of mouse E13.5, ferret E36, and human 11 wpc neocortex. To this end, we subtracted the internal standard-adjusted immunofluorescence signal for nuclear phospho-YAP from that of nuclear total YAP to obtain information about the relative levels of nuclear dephospho-YAP (see Figure S2B legend for details). This revealed substantially higher relative levels of dephospho-YAP in the Sox2-positive BP nuclei of ferret E36 and human 11 wpc neocortex than mouse E13.5 neocortex (Figure S2B).

We complemented these data by determining the effect of protein phosphatase treatment of cryosections on the YAP immunofluorescence signals in Sox2-positive BP nuclei in the SVZ of mouse E14.5, ferret E36, and human 12–13 wpc neocortex. For YAP immunofluorescence, two rabbit monoclonal antibodies were used together, one recognizing YAP irrespective of serine 127 (human) or serine 112 (mouse) phosphorylation (total YAP) and the other recognizing the serine 127 or serine 112 phosphorylation site when phosphorylated (phospho-YAP). Hence, in the control (i.e., without protein phosphatase treatment), the YAP immunofluorescence signal reflects the binding of primary antibodies to one or two sites, depending on whether serine 127 or serine 112 is phosphorylated or not. In the case of mouse, protein phosphatase treatment reduced the YAP immunofluorescence signal by ∼40% compared to control (Figure S2C). As protein phosphatase treatment of mouse E14.5 neocortex resulted in complete dephosphorylation of serine 112 (see STAR Methods), this ∼40% reduction suggested that approximately two-thirds (40/60) of the YAP protein in Sox2-positive BP nuclei of mouse E14.5 neocortex was in phosphorylated form. In contrast, in the case of ferret and human, protein phosphatase treatment reduced the YAP immunofluorescence signal by only ∼20% and 10%, respectively, compared to the controls (Figure S2C), consistent with only ∼25% (20/80) and ∼10% (10/90) of the YAP protein in the Sox2-positive BP nuclei of ferret E36 neocortex and human 12–13 wpc neocortex, respectively, being in phosphorylated form. These data therefore corroborated our finding that there are markedly higher relative levels of dephospho-YAP in the Sox2-positive BP nuclei of embryonic ferret and fetal human than embryonic mouse neocortex.

### Most Tbr2-Positive BPs in Embryonic Ferret and Fetal Human Neocortex Lack YAP Expression

Results essentially opposite to the occurrence of YAP in Sox2-positive BP nuclei were obtained when we compared the expression of YAP with that of Tbr2 (Figures 1F–1H), which in embryonic mouse neocortex has been established as a marker of differentiating BPs, notably of neurogenic bIPs (Englund et al., 2005, Kowalczyk et al., 2009, Pontious et al., 2008). Thus, only a minor proportion (<16%) of the Tbr2-positive nuclei in the E14.5 mouse, E36 ferret, and 11 wpc human SVZ were also positive for YAP (Figure 1K). Similarly, only ∼20% of the Tbr2-positive nuclei in the VZ of developing mouse, ferret, and human neocortex, which constitute newborn BPs (Arai et al., 2011), were YAP positive (Figure 1J). In contrast, the overwhelming majority (>75%) of the Tbr2-negative nuclei in the VZ of developing mouse, ferret, and human neocortex, which constitute APs capable of expansion by proliferation, were YAP positive (Figure 1I).

Taken together, these data suggest that the proliferative capacity of cNPCs, notably of BPs, correlates with the occurrence of nuclear dephospho-YAP, with a high percentage of proliferating APs in developing mouse, ferret, and human neocortex and proliferating BPs in embryonic ferret and fetal human neocortex exhibiting nuclear (and mostly active) YAP, whereas this was the case for only a low percentage of the proliferating BPs in embryonic mouse neocortex. Conversely, only a low percentage of the nuclei of differentiating (rather than proliferating) BPs in the developing mouse, ferret, and human neocortex exhibited nuclear YAP (and hence nuclear YAP activity, if any).

### Conditional Expression of Constitutively Active YAP in Embryonic Mouse Neocortex Decreases Tbr2-Positive BPs and Increases Sox2-Positive BPs

**Figure 2:**
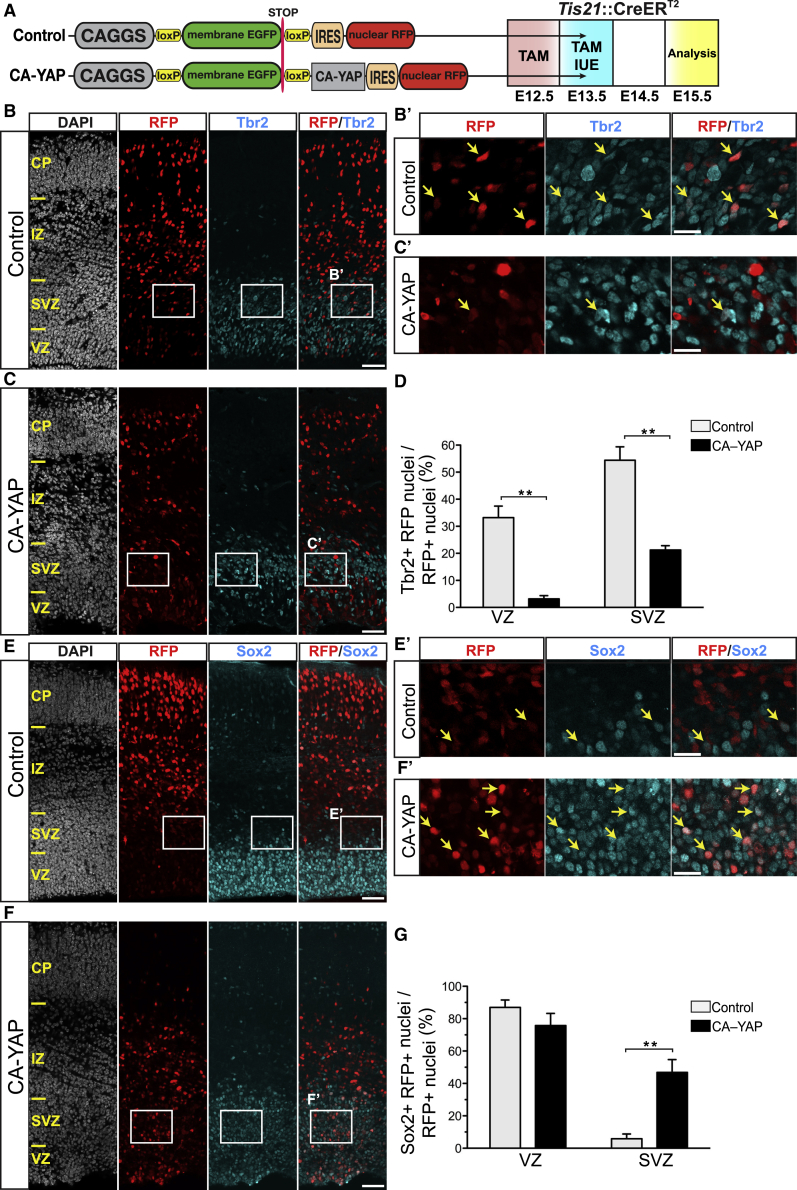
Conditional CA-YAP Expression in the BP-Genic Lineage of Embryonic Mouse Neocortex Decreases Production of Tbr2-Positive BPs and Increases Generation of Sox2-Positive BPs (A) Left: cartoon showing control (top) and CA-YAP-expressing (bottom) plasmid. Right: flow scheme of experiments. *Tis21*::CreER^T2^ heterozygous mouse embryos received tamoxifen (TAM) at E12.5 and E13.5, and the neocortex was subjected to IUE at E13.5 with control plasmid (B, D, E, and G) or CA-YAP-expressing plasmid (C, D, F, and G) followed by analysis at E15.5. (B, C, E, and F) Double immunofluorescence for RFP (red) and either Tbr2 (B and C) or Sox2 (E and F) (cyan), combined with DAPI staining (white). Boxes indicate areas in the SVZ that are shown at higher magnification (B′, C′, E′, and F′); arrows indicate selected RFP-positive nuclei that are Tbr2 positive (B′ and C′) or Sox2 positive (E′ and F′). Images are 1-μm optical sections. Scale bars represent 50 μm (B, C, E, and F) and 20 μm (B′, C′, E′, and F′). (D and G) Quantification of the percentage of RFP-positive nuclei that are Tbr2 positive (D) and Sox2 positive (G) in the VZ and SVZ upon control (light gray) and CA-YAP (black) electroporation. Two images (1-μm optical sections), each of a 200-μm-wide field of cortical wall, per embryo were taken, and the percentage values obtained were averaged for each embryo. Data are the mean of four embryos from four separate litters. The mean ± SEM is shown; ^∗∗^p < 0.01 (Mann-Whitney *U* test). See also Figures S3 and S5.

To examine if YAP activity is functionally linked to the proliferative capacity of BPs, we conditionally expressed a constitutively active YAP (CA-YAP) in BPs of the embryonic mouse neocortex, which lack proliferative potential and in which YAP expression normally is very low (Figures 1A, 1D, and 1E). In the CA-YAP, two serine residues were replaced by alanine (S112A and S382A), mutations that have been shown to stabilize and to increase the nuclear localization of the YAP protein (Camargo et al., 2007, Dong et al., 2007, Zhao et al., 2010). The plasmid used for CA-YAP expression (referred to as CA-YAP-expressing plasmid) contained a strong constitutive promoter (CAGGS) driving a membrane EGFP and a transcriptional stop sequence, flanked by *loxP* sites, followed by the CA-YAP and an internal ribosome entry site (IRES)-linked nuclear RFP reporter (Figure 2A, left). Expression of CA-YAP and RFP upon Cre-mediated excision of the floxed EGFP was validated by transfection of HEK293T cells (Figure S3A). The same plasmid but lacking the CA-YAP module was used as control (Figure 2A, left). Conditional expression of CA-YAP predominantly in mouse BPs was achieved by *in utero* electroporation (IUE) of the neocortex of E13.5 embryos of the *Tis21*-CreER^T2^ knockin (*Btg2*^*tm1.1(cre/ERT2)Wbh*^) mouse line (Wong et al., 2015) (Figure 2A right). In embryos of this mouse line, the expression of tamoxifen-activated Cre follows that of *Tis21*, which is specific for BP-genic aRG and BPs (Wong et al., 2015). We validated that, by analysis of E14.5 embryos, the conditional CA-YAP expression in mouse neocortex by this approach indeed occurred predominantly in BPs. Specifically, this analysis revealed that the overwhelming majority of the strongly YAP-expressing cells were BPs rather than APs (Figures S3B and S3C). Furthermore, conditional CA-YAP expression by this approach drove expression of a previously identified YAP target gene, CTGF (Malik et al., 2015, Zanconato et al., 2015, Zhao et al., 2008), in BPs 3 days after IUE (Figures S3B and S3D; for details, see Methods S1).

We first examined the effects of conditional CA-YAP expression on BP fate by analyzing Tbr2 and Sox2 expression 2 days after IUE of *Tis21*-CreER^T2^ mouse embryos (Figures 2A–2G). This revealed, among the RFP-positive progeny of the targeted cells, a marked reduction in the proportion of Tbr2-positive cells in the VZ and SVZ (Figure 2D) and a striking increase in the proportion of Sox2-positive cells in the SVZ, but not VZ (Figure 2G), compared to control. Hence, increasing YAP activity in neocortical BPs of embryonic mouse is sufficient to change the expression of transcription factors that are characteristic of either neuronal differentiation or proliferation, respectively.

### Conditional CA-YAP Expression in Embryonic Mouse Neocortex Increases the Proliferative Capacity of BPs

**Figure 3:**
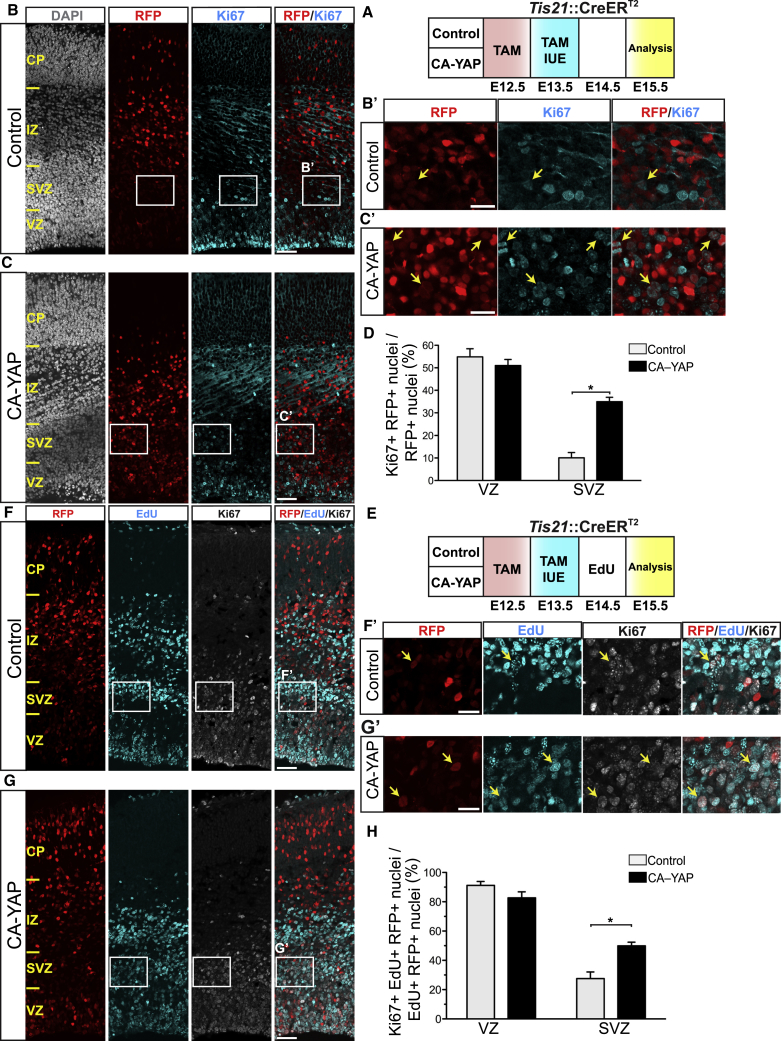
Conditional CA-YAP Expression in the BP-Genic Lineage of Embryonic Mouse Neocortex Promotes BP Proliferation and Cell Cycle Reentry (A–H) *Tis21*::CreER^T2^ heterozygous mouse embryos received tamoxifen (TAM) at E12.5 and E13.5, the neocortex was subjected to IUE at E13.5 with control plasmid (B, D, F, and H) or CA-YAP-expressing plasmid (C, D, G, and H) (see Figure 2A), embryos did not (B–D) or did (F–H) receive a single EdU pulse at E14.5, and the neocortex was analyzed at E15.5, as shown in the flow schemes of the experiments in (A) and (E), respectively. (B and C) Double immunofluorescence for RFP (red) and Ki67 (cyan), combined with DAPI staining (white). Boxes indicate areas in the SVZ that are shown at higher magnification in (B′) and (C′); arrows indicate selected RFP-positive nuclei that are Ki67 positive. (D) Quantification of the percentage of RFP-positive nuclei that are Ki67 positive in the VZ and SVZ upon control (light gray) and CA-YAP (black) electroporation. (F and G) Triple (immuno)fluorescence for RFP (red), EdU (cyan), and Ki67 (white). Boxes indicate areas in the SVZ that are shown at higher magnification (F′ and G′); arrows indicate selected RFP- and EdU-positive nuclei that are Ki67 positive. (H) Quantification of the percentage of RFP- and EdU-positive nuclei that are Ki67 positive in the VZ and SVZ upon control (light gray) and CA-YAP (black) electroporation. (B, C, F, and G) Images are 1-μm optical sections. Scale bars represent 50 μm (B, C, F, and G) and 20 μm (B′, C′, F′, and G′). (D and H) Two images (1-μm optical sections), each of a 200-μm-wide field of cortical wall, per embryo were taken, and the percentage values obtained were averaged for each embryo. Data are the mean of four embryos from four separate litters. The mean ± SEM is shown; ^∗^p < 0.05 (Mann-Whitney *U* test). See also Figures S3–S5.

Next, we directly examined the potential effects of conditional CA-YAP expression in embryonic mouse neocortex on BP proliferation using three distinct approaches. First, we performed immunofluorescence for the cycling cell marker Ki67 2 days (Figures 3A–3C) and 3 days (Figures S4A–S4C) after IUE. Conditional CA-YAP expression increased the proportion of Ki67-positive cells among the RFP-positive progeny of the targeted cells in the SVZ 2- to 3-fold (Figures 3D and S4D). Concomitant with this effect, conditional CA-YAP expression resulted in an altered distribution of the RFP-positive cells across the various zones of the cortical wall (Figures S5A–S5C), with a greater relative proportion of these cells in the VZ and a lesser relative proportion in the IZ and CP (Figure S5D). In terms of absolute progenitor cell numbers, conditional CA-YAP expression caused a more than 2-fold increase in Ki67- and RFP-positive cells in the VZ and SVZ (Figure S5E). These data raise the possibility of a relationship between the CA-YAP-induced progenitor proliferation and the reduced migration of the RFP-positive progeny of the targeted cells beyond the germinal zones to the basal region of the cortical wall.

Second, we performed immunofluorescence for the mitotic cell marker phosphovimentin (pVIM) (Figures S4A, S4E, and S4F). Conditional CA-YAP expression markedly increased the proportion of pVIM-positive cells among the RFP-positive progeny of the targeted cells in the SVZ (Figure S4G). Consistent with this, conditional CA-YAP expression also markedly increased the abundance of mitotic, pVIM-positive BPs (sum of RFP-positive and RFP-negative basal mitoses) (Figure S4H). Analysis of the pVIM- and RFP-positive cells remaining in the SVZ three days after IUE (Figure S4A) for the presence versus absence of pVIM-positive cell processes (typically a basal process) revealed no significant difference between the control and conditional CA-YAP expression (Figure S4I).

Third, we carried out a cell-cycle reentry assay. To this end, we performed a 5′-ethynyl-2′deoxyuridine (EdU) pulse labeling 24 h after IUE, followed by Ki67 immunofluorescence 24 h later (Figure 3E). Given the cell-cycle parameters of *Tis21*-GFP-positive APs and BPs at this stage of embryonic mouse neocortex development (Arai et al., 2011), the latter time interval is sufficient for the incorporated EdU to become inherited by daughter cells. Thus, EdU- and RFP-positive cells that are also positive for Ki67 are daughter cells that reentered the cell cycle (Figure 3E). Using this assay, we found that conditional CA-YAP expression doubled the cell-cycle reentry of BPs in the SVZ (Figures 3E–3H). Taken together, the results of these three lines of investigation demonstrate that conditional CA-YAP expression in embryonic mouse neocortex increases BP proliferation.

### Conditional CA-YAP Expression in Embryonic Mouse Neocortex Results in Reduced Deep-Layer Neuron and Increased Upper-Layer Neuron Generation

**Figure 4:**
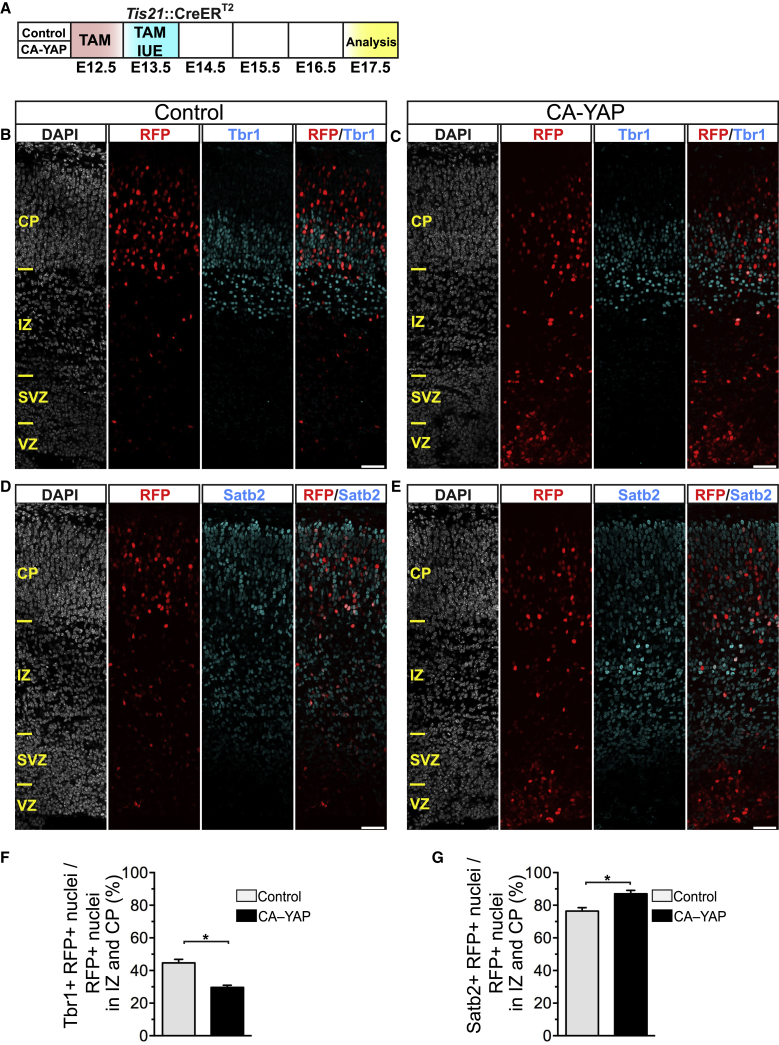
Conditional CA-YAP Expression in the BP-Genic Lineage of Embryonic Mouse Neocortex Decreases the Production of Deep-Layer Neurons and Increases the Production of Upper-Layer Neurons (A) Flow scheme of experiments. *Tis21*::CreER^T2^ heterozygous mouse embryos received tamoxifen (TAM) at E12.5 and E13.5, and the neocortex was subjected to IUE at E13.5 with control plasmid (B, D, F, and G) or CA-YAP-expressing plasmid (C and E–G) (see Figure 2A), followed by analysis at E17.5. (B–E) Double immunofluorescence for RFP (red) and either Tbr1 (B and C) or Satb2 (D and E) (cyan), combined with DAPI staining (white). Images are 1-μm optical sections. Scale bars, 50 μm. (F and G) Quantification of the percentage of RFP-positive nuclei that are Tbr1 positive (F) and Satb2 positive (G) in the intermediate zone (IZ) and cortical plate (CP) upon control (light gray) and CA-YAP (black) electroporation. Two images (1-μm optical sections), each of a 200-μm-wide field of cortical wall, per embryo were taken, and the percentage values obtained were averaged for each embryo. Data are the mean of four embryos from four separate litters. The mean ± SEM is shown; ^∗^p < 0.05 (Mann-Whitney *U* test). See also Figure S6.

Given that BPs generate most cortical neurons (Florio and Huttner, 2014, Lui et al., 2011), we explored the potential consequences of the CA-YAP-induced increase in BP proliferation for neuron generation in the embryonic mouse neocortex. To this end, we performed immunostaining for Tbr1, a deep-layer neuron marker, and Satb2, an upper-layer neuron marker, 4 days after IUE (Figures 4A–4E). Conditional CA-YAP expression reduced the proportion of Tbr1-positive neurons among the RFP-positive progeny of the targeted cells in the IZ and CP to almost half of control (29% versus 44%; Figure 4F) and caused a small but statistically significant increase in the proportion of Satb2-positive neurons (from 76% to 87%; Figure 4G).

Considering that the pool size of the RFP-positive progeny in the IZ and CP 4 days upon IUE (E13–E17) is not much affected by the conditional CA-YAP expression compared to control (Figure S6), these data are consistent with the notion that the changes in the proportions of specific types of neurons among the RFP-positive progeny reflect the changes in neuron generation in embryonic mouse neocortex. Specifically, on the one hand, the CA-YAP-induced increase in BP proliferation initially results in a reduced production of deep-layer neurons, because upon conditional CA-YAP expression, a certain proportion of mouse BPs have been induced to undergo symmetric proliferative divisions and hence a lesser proportion of mouse BPs are available to undergo the symmetric consumptive divisions that generate neurons. On the other hand, the CA-YAP-induced increase in BP proliferation eventually results in an increased mouse BP pool size that at later stages of cortical neurogenesis gives rise to more upper-layer neurons.

### Pharmacological Inhibition of YAP Activity Reduces Mitotic BP Abundance in Embryonic Ferret and Fetal Human Neocortex

**Figure 5:**
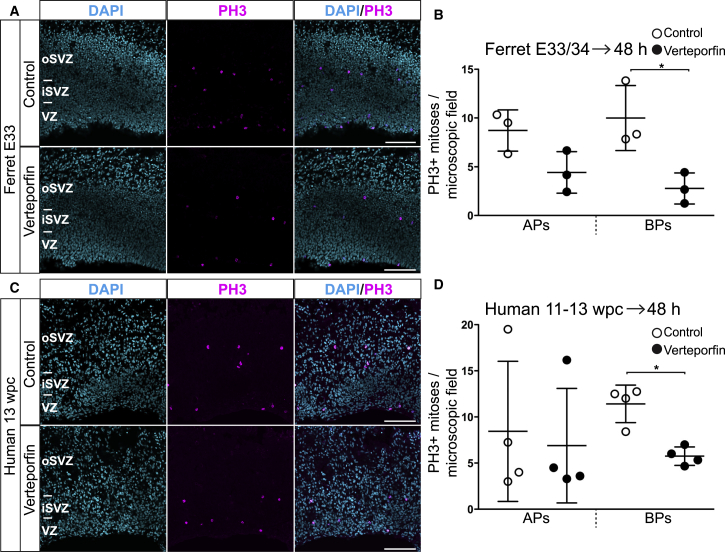
Inhibition of YAP Activity by Verteporfin Reduces Mitotic BP Levels in Embryonic Ferret and Fetal Human Neocortex (A–D) Ferret E33–E34 (A and B) and human 11–13 wpc (C and D) neocortex was incubated for 48 h in FFT culture in the absence (control, top rows in A and C and open circles in B and D) or presence (bottom rows in A and C and filled circles in B and D) of 1 μM verteporfin, followed by analysis. (A and C) Immunofluorescence for PH3 (magenta), combined with DAPI staining (blue), upon control (top rows) and verteporfin (bottom rows) treatment. Images are 1-μm optical sections. Scale bars, 100 μm. (B and D) Quantification of the number of APs and BPs in mitosis, as revealed by PH3 immunofluorescence, per microscopic field (400-μm-wide field of cortical wall), upon control (open circles) and verteporfin (filled circles) treatment. Five to eight images (1-μm optical sections) per either ferret embryo (B) or human fetus (D) were taken, and the values obtained were averaged for each embryo-fetus. Data are the mean of three ferret embryos from three separate litters (B) and of four human fetuses (D). Error bars indicate SD; ^∗^p < 0.05 (Mann-Whitney *U* test).

Having established that mimicking a ferret- or human-like expression of active YAP in BPs of embryonic mouse (i.e., lissencephalic) neocortex suffices to increase their proliferation, we next investigated whether the presence of active YAP in BPs of developing ferret and human (i.e., gyrencephalic) neocortex is a necessary requirement for their proliferation. To this end, we applied an inhibitor of YAP, verteporfin (Brodowska et al., 2014, Liu-Chittenden et al., 2012, Song et al., 2014, Wang et al., 2015), in an *ex vivo* free-floating tissue (FFT) culture system (Schenk et al., 2009) using E33–E34 ferret and 11–13 wpc human neocortical tissue (Figure 5). Verteporfin is a small molecule that inhibits the association of YAP with TEAD transcription factors and thereby prevents the expression of genes linked to cell proliferation (Liu-Chittenden et al., 2012).

Treatment of embryonic ferret and fetal human neocortical FFT cultures with 1 μM verteporfin for 48 h decreased the abundance of basal mitoses, as identified by phospho-histone H3 (PH3) immunofluorescence (Figures 5A and 5C), ∼4-fold and 2-fold, respectively (Figures 5B and 5D). The time period of 48 h corresponds roughly to the length of one cell cycle of BPs in ferret (Turrero García et al., 2016) and human (Hansen et al., 2010, Mora-Bermúdez et al., 2016) neocortex, and BPs were quantified at mitosis (i.e., at the end of their cell cycle). The verteporfin data therefore imply that the decrease in BP abundance must have occurred largely in the BPs themselves (as opposed to in APs that then gave rise to BPs).

Upon verteporfin treatment of embryonic ferret and fetal human neocortical FFT cultures, mitotic AP levels did not show a statistically significant decrease (Figures 5B and 5D), although in the case of ferret, the trend of a decrease could be observed (Figure 5B). A possible explanation why there was no decrease in human mitotic AP levels upon verteporfin treatment may be that the length of the human AP cell cycle did not allow manifestation of a decrease in mitotic AP levels within the 48-h period between addition of verteporfin and analysis.

### Dominant-Negative YAP Expression Reduces Mitotic BP Abundance in Embryonic Ferret Neocortex

We sought to obtain corroborating *in vivo* evidence for an essential role of YAP activity in BP proliferation in developing gyrencephalic neocortex. To this end, we used a dominant-negative YAP construct (DN-YAP) to block its transcriptional co-activator function (Nishioka et al., 2009, Sudol et al., 2012) (see STAR Methods for details). In this DN-YAP construct, the transactivation domain of mouse YAP is replaced with the engrailed domain, a *Drosophila* repressor of transcription (Nishioka et al., 2009), and nuclear localization of DN-YAP is ensured by replacing YAP serine 112 with alanine (Hao et al., 2008, Zhao et al., 2007). Forced expression of DN-YAP creates multiple copies of DN-YAP that outcompete endogenous wild type YAP (wtYAP) in binding to TEAD transcription factors, which causes downregulation of YAP-driven genes linked to proliferation (Nishioka et al., 2009, Zanconato et al., 2015). We chose embryonic ferret neocortex to examine the effects of DN-YAP expression on BP proliferation.

**Figure 6:**
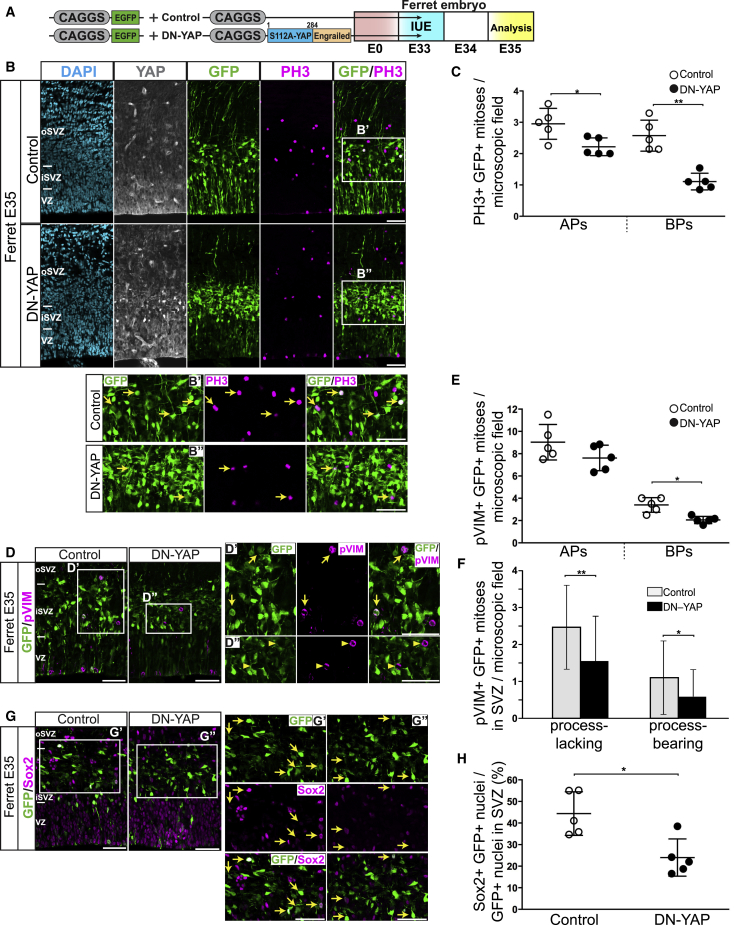
Expression of a DN-YAP in Embryonic Ferret Neocortex Reduces Mitotic BP Levels (A) Left: cartoon showing the EGFP-expressing plasmid plus the control plasmid (top) and the EGFP-expressing plasmid plus the DN-YAP-expressing plasmid (bottom). (B–H) Right: flow scheme of experiment. Ferret neocortex was electroporated at E33 with either GFP-expressing plus control plasmids (B, top row; B′, C, and D, left; D′ and E–G, left; and G′ and H) or GFP-expressing plus DN-YAP-expressing plasmids (B, bottom row; B″, C, and D, right; D″ and E–G, right; and G″ and H), followed by analysis at E35. (B) Triple immunofluorescence for YAP (white), GFP (green), and PH3 (magenta), combined with DAPI staining (blue). Boxes indicate areas in the SVZ that are shown at higher magnification in B′ and B″; arrows indicate selected GFP-positive cells that are PH3 positive. (C) Quantification of the number of GFP-positive APs and BPs in mitosis, as revealed by PH3 immunofluorescence, per microscopic field (200-μm-wide field of cortical wall) upon control (open circles) and DN-YAP (filled circles) electroporation. 6–15 images (1-μm optical sections) per ferret embryo were taken, and the values obtained were averaged for each embryo. The data show the averaged values for five ferret embryos from one litter. (D) Double immunofluorescence for GFP (green) and pVIM (magenta). Boxes indicate areas in the SVZ that are shown at higher magnification (D′ and D″); arrows in (D′) indicate selected GFP-positive cells that are pVIM positive; arrowheads in (D″) indicate selected pVIM-positive nuclei that are GFP negative. (E) Quantification of the number GFP-positive APs and BPs in mitosis, as revealed by pVIM immunofluorescence, per microscopic field (200-μm-wide field of cortical wall) upon control (open circles) and DN-YAP (filled circles) electroporation. Six images (each a 5-μm z stack) per ferret embryo were taken, and the values obtained were averaged for each embryo. The data show the averaged values for five ferret embryos from one litter. (F) Quantification of the number of process-lacking (left) and process-bearing (right) GFP-positive mitoses in the SVZ, as revealed by pVIM immunofluorescence, per microscopic field (200 μm-wide field of cortical wall) upon control (light gray) and DN-YAP (black) electroporation. Data are the mean of multiple fields counted (control, 30; DN-YAP, 29) from five ferret embryos. (G) Double immunofluorescence for GFP (green) and Sox2 (G) (magenta). Boxes indicate areas in the SVZ that are shown at higher magnification (G′ and G″); arrows indicate selected GFP-positive nuclei that are Sox2 positive. (H) Quantification of the percentage of GFP-positive cells in the SVZ that are Sox2 positive upon control (open circles) and DN-YAP (filled circles) electroporation. Six images (1-μm optical sections), each of a 200-μm-wide field of cortical wall, were taken per ferret embryo, and the percentage values obtained were averaged for each embryo. Data are the mean of five ferret embryos from one litter. (B, B′, B″, D, D′, D″, G, G′, and G″) Images are 1-μm optical sections. Scale bars, 50 μm. (C, E, and H) The mean ± SD is shown; ^∗^p < 0.05, ^∗∗^p < 0.01 (Mann-Whitney *U* test).

We performed IUE of ferret embryos to deliver the DN-YAP construct into the developing ferret neocortex (Kawasaki et al., 2012, Kawasaki et al., 2013). Specifically, we co-electroporated the ferret dorsolateral neocortex with either CAGGS-empty vector plus CAGGS-EGFP or with CAGGS-DN-YAP plus CAGGS-EGFP. IUE was performed at E33, the stage that corresponds to mid-neurogenesis in the ferret. Ferret embryos were harvested 2 days later, at E35, and mitotic BPs were quantified. Given the length of the cell cycle of ferret BPs (Turrero García et al., 2016), the 2-day period between IUE and analysis (Figure 6A) should suffice for the targeted BPs to complete their cell cycle and enter mitosis. To confirm the expression of DN-YAP, we performed immunostaining using a YAP antibody that recognizes both endogenous YAP and DN-YAP and compared the level of YAP immunoreactivity in GFP-positive cells upon DN-YAP expression to that of control. Upon DN-YAP expression, many of the GFP-positive cells showed a higher level of YAP immunoreactivity, especially in the SVZ, suggesting that the DN-YAP was successfully expressed in BPs (Figure 6B; compare bottom and top rows).

Analysis of GFP-positive mitoses, identified by PH3 immunofluorescence, revealed that expression of DN-YAP drastically decreased the abundance of mitotic BPs (Figures 6B and 6C). Similar results were obtained by quantitation of GFP- and pVIM-positive basal mitoses (Figures 6D and 6E). In contrast, DN-YAP expression caused only a small decrease in the abundance of GFP- and PH3-positive mitotic APs (Figures 6B and 6C) and no statistically significant decrease in the abundance of GFP- and pVIM-positive mitotic APs (Figures 6D and 6E).

There are two main types of BPs: (1) those lacking processes at mitosis (i.e., bIPs) and (2) those bearing radial processes at mitosis (i.e., bRG) (Fietz et al., 2010, Kelava et al., 2012, Reillo et al., 2011). To examine whether any of these two types of BPs are preferentially affected by DN-YAP expression, we analyzed the processes as revealed by pVIM staining in GFP-positive mitotic BPs in the SVZ. We observed a similar decrease in process-lacking and process-bearing BPs (Figure 6F), which suggests that the effect of DN-YAP expression is nonselective with regard to the BP population type, affecting equally bIPs and bRG.

Given that conditional CA-YAP expression in embryonic mouse neocortex resulted in a specific increase in Sox2-positive BPs (Figure 2G), we explored whether inhibition of YAP activity in embryonic ferret neocortex would affect the Sox2-positive pool of BPs in the SVZ. Indeed, DN-YAP expression reduced the proportion of Sox2-positive BPs among the GFP-positive progeny of the targeted cells in the SVZ (Figures 6G and 6H).

### Disruption of *YAP* Expression in Fetal Human Neocortex Reduces BP Abundance

**Figure 7:**
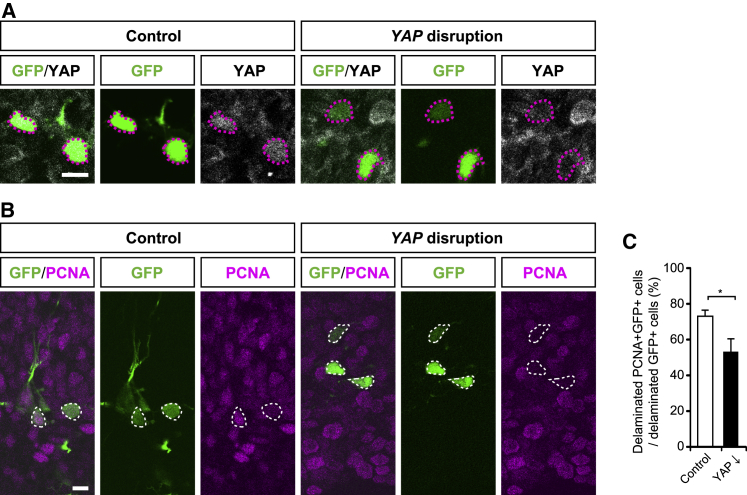
CRISPR-Cas9-Mediated Disruption of *YAP* Expression in Fetal Human Neocortical Tissue Reduces BP Abundance (A–C) Human 12- to 14-wpc neocortical tissue was electroporated *ex vivo* with either GFP-expressing plus control plasmids (A and B, left; and C) or a GFP-expressing plus *YAP*-disrupting plasmid (see STAR Methods) (A and B, right; and C) and then incubated for 72 h in FFT culture, followed by analysis. (A) Double immunofluorescence for GFP (green) and YAP (white) upon control electroporation (left) and *YAP* gene disruption (right). Delaminated cells, as identified by GFP immunofluorescence, that exhibit YAP expression in the control condition (left) and show reduced YAP expression upon *YAP* gene disruption (right) are indicated by red dotted lines. Images are 1.2-μm optical sections. Scale bar, 10 μm. (B) Double immunofluorescence for GFP (green) and PCNA (magenta) upon control electroporation (left) and *YAP* gene disruption (right). Delaminated cells, as identified by GFP immunofluorescence, that exhibit PCNA expression in the control condition (left) and show reduced PCNA expression upon *YAP* gene disruption (right) are indicated by white dashed lines. Images are 1.2-μm optical sections. Scale bar, 10 μm. (C) Quantification of the percentage of delaminated GFP+ cells that are overtly PCNA positive (PCNA^+^) upon control electroporation (open bar) and *YAP* gene disruption (YAP ↓, solid bar). Control: data are the mean of four electroporated tissues (n = 4) from two fetus (14 wpc and 12 wpc), with a total of 88 delaminated GFP+ cells counted. For YAP ↓, data are the mean of three electroporated tissues (n = 3) from three fetus (14 wpc, 13 wpc, and 13 wpc), with a total of 118 delaminated GFP+ cells counted. Error bars indicate SEM; ^∗^p < 0.05 (non-paired Student’s t test).

We complemented the data obtained upon DN-YAP expression in embryonic ferret neocortex by disrupting the expression of the *YAP* gene in fetal human neocortical tissue using CRISPR-Cas9 technology. Human neocortical tissue from 12 to 14 wpc fetuses was electroporated with either CAGGS-EGFP plus a CRISPR-Cas9 control plasmid or a GFP-expressing plus *YAP*-disrupting plasmid. Analyses after 72 h in FFT culture revealed that upon disruption of *YAP* expression, many of the targeted delaminated cells, as identified by GFP immunofluorescence, showed a lower level of YAP immunoreactivity than control delaminated cells (Figure 7A). This allowed us to compare the proportion of cycling cells, identified by proliferating cell nuclear antigen (PCNA) immunofluorescence, among the targeted delaminated (i.e., GFP+) cells (that is, BP abundance) upon control electroporation and disruption of *YAP* expression (Figure 7B). This comparison revealed that the disruption of *YAP* expression significantly decreased BP abundance (Figure 7C). Hence, YAP expression in BPs is required to maintain their normal level in fetal human neocortex.

Taken together, our data using verteporfin administration, DN-YAP expression, and disruption of *YAP* expression indicate that YAP activity is necessary for the proliferation of BPs in developing gyrencephalic neocortex *in vivo*.

Our observation that YAP expression and Tbr2 expression in ferret and human BPs are mutually exclusive (Figure 1K) precluded the development of a BP-specific YAP knockout approach based on the specificity of the *Eomes*-*EOMES* promoter.

## Discussion

The present study demonstrates a crucial role of YAP, the central effector of the Hippo signaling pathway, in the development and evolution of the mammalian neocortex. Specifically, our findings advance our understanding of cNPC activity in cortical development and its differences across mammals with regard to two key aspects. First, YAP activity is shown to be necessary and sufficient to maintain the proliferative capacity of BPs at levels typically seen in developing gyrencephalic neocortex. Second, differences in YAP activity in BPs between embryonic mouse, which develops a lissencephalic neocortex, on the one hand, and embryonic ferret and fetal human, which develop a gyrencephalic neocortex, on the other hand, are shown to contribute to, if not underlie, the differences in BP proliferative capacity across these species. Three aspects of our findings deserve particular consideration.

### Differential YAP Expression between the VZ and SVZ across Species

Previous reports on the role of YAP were confined to mouse brain development and concentrated on APs (Cappello et al., 2013, Lavado et al., 2013, Lavado et al., 2014, Saito et al., 2018). In contrast to these reports, the present study is focused on BPs and in this context has compared YAP expression and activity in the SVZ versus VZ of developing neocortex of three mammals exhibiting a different extent of neocortex expansion: mouse, ferret, and human. Our demonstration that in species in which BPs exhibit significant proliferative capacity, YAP expression and activity are well detectable not only in the VZ but also in the SVZ has at least two implications. First, to the best of our knowledge, these data constitute the first report of YAP protein expression in the iSVZ and oSVZ of developing neocortex. Second, the present findings suggest that the role of YAP, and hence Hippo signaling, in the proliferation and consequently the pool size of cNPCs is far more widespread than previously assumed.

### YAP Is a Putative Downstream Target of Sox2 in BPs

The co-expression of active YAP with Sox2 in ferret and human BPs suggests an interesting mechanistic scenario regarding the regulation of YAP expression and, consequently, activity. Recently, it was shown that Sox2 directly drives the expression of YAP in progenitors of the osteo-adipo lineage (Seo et al., 2013). Given that mouse BPs exhibit lower Sox2 expression than mouse APs whereas ferret and human BPs maintain a Sox2 expression similar to APs (Figures 1A–1C), our results raise the possibility that YAP expression in ferret and human proliferative BPs is driven by Sox2. Furthermore, while the ability of YAP to activate transcription is known to be decreased by phosphorylation via upstream Hippo signaling kinases such as Nf2 and Wwc1 (Yu et al., 2015), it was recently reported that in cancer stem cells, Sox2 represses transcription of the *NF2* and *WWC1* genes, which in turn increased YAP activity (Basu-Roy et al., 2015). Our finding of increased YAP activity in proliferative BPs of developing gyrencephalic neocortex could therefore be explained by Sox2 in these cells repressing *NF2* and *WWC1*.

### BP Fate Switch upon Conditional YAP Expression

However, our data not only are consistent with the notion that in ferret and human BPs Sox2 positively regulates YAP expression levels and activity but also indicate that forced YAP expression in mouse BPs, directly or indirectly, results in increased Sox2 expression and decreased Tbr2 expression. In other words, the present approach of conditionally expressing CA-YAP in the mouse Tis21-positive BP lineage induced a BP fate switch from neurogenic to proliferative. This in turn led to an expansion of the BP pool, which eventually resulted in an increased generation of upper-layer neurons. Hence, increasing YAP activity in BPs is sufficient to induce features that are hallmarks of an expanded neocortex, as characteristically observed in gyrencephalic mammals (Florio and Huttner, 2014, Lui et al., 2011). This, together with our finding that inhibiting YAP activity in BPs of developing ferret and human neocortex reduced their abundance, in turn leads us to conclude that an increase in YAP activity in BPs of developing neocortex likely was a major contributor to its evolutionary expansion.

## STAR★Methods

### Key Resources Table

**Table:** 

REAGENT or RESOURCE	SOURCE	IDENTIFIER
**Antibodies**		

Goat polyclonal anti-CTGF (1:100)	Santa Cruz Biotechnology	Cat# sc-14939; RRID:AB_638805
Chicken polyclonal anti-GFP (1:500)	Aves	Cat# GFP-1020; RRID:AB_10000240
Rabbit polyclonal anti-Ki67 (1:100)	Abcam	Cat# ab15580; RRID:AB_443209
Rat monoclonal anti-PH3-S28 (1:250)	Abcam	Cat# ab10543; RRID:AB_2295065
Mouse monoclonal anti-PCNA (1:500)	Millopore	Cat# CBL407; RRID:AB_93501
Mouse monoclonal anti-pVIM (1:100)	Abcam	Cat# ab22651; RRID:AB_447222
Rat monoclonal anti-RFP (1:500)	ChromoTek	Cat# 5F8; RRID: AB_2336064
Goat polyclonal anti-Sox2 (1:100)	Santa Cruz Biotechnology	Cat# sc-17320; RRID:AB_2286684
Goat polyclonal anti-Sox2 (1:100)	R&D Systems	Cat# AF2018; RRID:AB_355110
Rabbit polyclonal anti-Tbr1 (1:100)	Abcam	Cat# ab31940; RRID:AB_2200219
Rabbit polyclonal anti-Tbr2 (1:200)	Abcam	Cat# ab23345; RRID:AB_778267
Mouse monoclonal anti-Tbr2 (1:50)	MPI-CBG, This paper	N/A
Rabbit polyclonal anti-phospho-YAP-S127 (1:50)	Cell Signaling	Cat# 4911; RRID:AB_2218913
Rabbit monoclonal anti-phospho-YAP-S127 (1:100)	Cell Signaling	Cat# 13008; RRID:AB_2650553
Rabbit monoclonal anti-YAP (1:100)	Cell Signaling	Cat# 14074; RRID:AB_2650491
Mouse monoclonal anti-YAP (1:100)	Abcam	Cat# ab56701; RRID:AB_2219140
Donkey anti-Goat IgG (H+L) Alexa Fluor 647 (1:500)	Thermo Fisher Scientific	Cat# A-21447; RRID:AB_141844
Goat anti-Chicken IgY (H+L) Alexa Fluor 488	Thermo Fisher Scientific	Cat# A-11039; RRID: AB_142924
Donkey anti-Rabbit IgG (H+L), Alexa Fluor 488 (1:500)	Thermo Fisher Scientific	Cat#R37118; RRID:AB_2556546
Donkey anti-Rabbit IgG (H+L), Alexa Fluor 555 (1:500)	Thermo Fisher Scientific	Cat# A-31572; RRID:AB_162543
Donkey anti-Rabbit IgG (H+L), Alexa Fluor 647 (1:500)	Thermo Fisher Scientific	Cat# A-31573; RRID: AB_2536183
Donkey anti-Mouse IgG (H+L), Alexa Fluor 488 (1:500)	Thermo Fisher Scientific	Cat# R37114; RRID: AB_2556542
Donkey anti-Mouse IgG (H+L), Alexa Fluor 555 (1:500)	Thermo Fisher Scientific	Cat# A-31570; RRID: AB_2536180
Donkey anti-Mouse IgG (H+L), Alexa Fluor 647 (1:500)	Thermo Fisher Scientific	Cat# A-31571; RRID: AB_162542
Donkey anti-Rat IgG (H+L) Cy3 (1:500)	Jackson ImmunoResearch	Cat# 712-185-153; RRID: AB_2340667
Donkey anti-Rat IgG (H+L) Cy5 (1:500)	Jackson ImmunoResearch	Cat# 712-175-153; RRID: AB_2340672

**Biological Samples**		

Human fetal brain tissue	Klinik und Poliklinik für Frauenheilkunde und Geburtshilfe, Universitätsklinikum Carl Gustav Carus of the Technische Universität Dresden	N/A
Human fetal brain tissue	Human Developmental Biology Resource (www.hdbr.org)	N/A

**Chemicals, Peptides, and Recombinant Proteins**		

Tamoxifen	Sigma	Cat# T-5648
Verteporfin	Sigma	Cat# SML0534
Lambda protein phosphatase	NEB	Cat# P0753S
		N/A

**Experimental Models: Cell Lines**		

HEK293T		N/A

**Experimental Models: Organisms/Strains**		

Mouse: C57BL/6JOlaHsd	Envigo	N/A
Mouse: *Tis21*-CreER^T2^	Wong et al., 2015	N/A
Ferret	Marshall Bioresourcesm North Rose, NY, USA	N/A

**Critical Commercial Assays**		

Maxi prep kit	QIAGEN	Cat# 12362
Click-it™ Edu Alexa Fluor™ 647 imaging kit	Thermo Fisher Scientific	Cat# C10340

**Oligonucleotides**		

Primer: YAP-SalI-F: 5′-gcgcgtcgacgccaccatggagccc gcgcaacagcc-3′	This paper	N/A
Primer: YAP-SalI-R: 5′-cgcggtcgacctataaccacgtgagaaagct-3′	This paper	N/A
Primer: S112A-F: 5′-catgttcgagctcacgcctctccagcctccc-3′	This paper	N/A
Primer: S112A-R: 5′-gggaggctggagaggcgtgagctcgaacatg-3′	This paper	N/A
Primer: S382A-F: 5′-cactctcgagatgaggccacagacagcggcc-3′	This paper	N/A
Primer: S382A-R: 5′-ggccgctgtctgtggcctcatctcgagagtg-3′	This paper	N/A
Primer: BamHI-HA-F: 5′-gcgcggatccgccaccatgtaccc atacgacgttcc-3′	This paper	N/A
Primer: BamHI-HA-R: 5′-cgcgggatccctagttcaggtcctcctcgg-3′	This paper	N/A

**Recombinant DNA**		

Mouse wtYAP isoform 1 cDNA clone	Source Bioscience	IMAGE 4239820
pCR2.1-TOPO-TA vector	ThermoFisher Scientific	K450002
pTOPO-mouse-wtYAP	This paper	N/A
pCAGGS-LoxP-Gap43-GFP-LoxP-IRES-nRFP	Wong et al., 2015	N/A
pCAGGS-LoxP-Gap43-YAP-LoxP-IRES-nRFP	This paper	N/A
pCAGGS-LoxP-Gap43-YAP-S112A-S382A-LoxP-IRES-nRFP	This paper	N/A
pcDNA3.1-pA83-dnYAP	RIKEN BRC (Nishioka et al., 2009)	Cat# RDB12195
pCAGGS-empty	Niwa et al., 1991	N/A
pCAGGS-DN-YAP	This paper	N/A
LacZ CRISPR/Cas9 Plasmid	Kalebic et al., 2016	N/A
YAP CRISPR/Cas9 KO Plasmid	Santa Cruz Biotechnology	sc-400040

**Software and Algorithms**		

Excel 2016	Microsoft, Redmond, WA	N/A
Prism 5	GraphPad Software, Inc	RRID: SCR_002798
Fiji v2.0.0.-rc-43/1.51e	Schindelin et al., 2012	RRID: SCR_002285

### Contact for Reagent and Resource Sharing

Further information and requests for resources and reagents should be directed to and will be fulfilled by the Lead Contact, Wieland Huttner (huttner@mpi-cbg.de).

### Experimental Model and Subject Details

#### Ethics

All animal experiments (mice and ferrets) were performed in accordance with the German Animal Welfare legislation (“Tierschutzgesetz”). All procedures regarding the animal experiments were approved by the Governmental IACUC (“Landesdirektion Sachsen”) and overseen by the Institutional Animal Welfare Officer(s). The license numbers concerning the experiments with mice are: Untersuchungen zur Neurogenese in Mäuseembryonen TVV2015/05 (in utero electroporation, tamoxifen, EdU) and 24–9168.24-9/2012-1 (tissue collection without prior *in vivo* experimentation). The license number concerning the experiments with ferrets is: Untersuchungen zur Neurogenese in Frettchen” (TVV 2015/02) issued by “Landesdirektion Sachsen.”

#### Mice

To characterize YAP expression E13-14 mouse embryos (C57BL/6JOlaHsd) were used. The sex of embryos was not determined because the male versus female phenotype is not yet fully developed at the developmental stage concerned. For electroporation E13 *Tis21*-CreER^T2+/−^ heterozygous mouse embryos were used. These were obtained by crossing C57BL/6JOlaHsd females and *Tis21*-CreER^T2+/+^ males (Wong et al., 2015). Mice were crossed and kept under strict pathogen-free conditions in the animal facility of the Max Planck Institute of Molecular Cell Biology and Genetics.

#### Ferrets

Normally pigmented pregnant female sable ferrets (*Mustela putorius furo*) were purchased from Marshall BioResources (North Rose, NY, USA). They were delivered and housed in the animal facility of the Max Planck Institute of Molecular Cell Biology and Genetics. In utero electroporation and verteporfin treatment was performed on E33 embryos. The sex of embryos was not determined because the male versus female phenotype is not yet fully developed at the developmental stage concerned.

#### Human fetal tissue

Human fetal brain tissue was obtained from two sources. First, from the Klinik und Poliklinik für Frauenheilkunde und Geburtshilfe, Universitätsklinikum Carl Gustav Carus of the Technische Universität Dresden, following elective pregnancy termination and informed written maternal consents (see Methods S1), and with approval of the local University Hospital Ethical Review Committees. The age of a 12 wpc fetus (n = 1) was assessed by ultrasound measurements of crown-rump length and other standard criteria of developmental stage determination. The second source was the Human Developmental Biology Resource (HDBR). This human fetal brain tissue was provided by the Joint MRC/Wellcome Trust (grant # MR/R006237/1) Human Developmental Biology Resource (www.hdbr.org). The HDBR provided fresh tissue from fetuses aged 11-14 wpc, (11 wpc, n = 4; 12 wpc, n = 3; 13 wpc, n = 4; wpc 14, n = 2). Due to protection of data privacy neither gender identity nor sex of the human fetuses of which neocortex tissue was obtained can be reported. Neither gender identity nor sex of the human fetuses is likely to be of relevance for the results obtained in the present study. Human fetal brain tissue was dissected in 1x PBS and used immediately for culture or fixation (as indicated) when obtained from Dresden. When obtained from HDBR, tissue was dissected and shipped in Hibernate E media (GIBCO A1247601). Upon arrival, all tissue was cultured in slice culture medium (SCM, see section on *ex vivo* FFT cultures) for 2-3 h prior to any further manipulation. All tissue was fixed for at least 24 h at 4°C in 4% paraformaldehyde in 120 mM phosphate buffer (pH 7.4) (referred to in short as 4% PFA).

### Method Details

#### YAP DNA constructs

Mouse wtYAP isoform 1 cDNA clone (IMAGE 4239820) was obtained from Source Bioscience. Restriction enzyme sites, SalI, were adapted to the 5′ and 3′ ends of wtYAP by PCR. wtYAP was digested by SalI and subcloned to the *pCR2.1-TOPO-TA* vector (Invitrogen), creating an intermediate, non-expressing vector *pTOPO-mouse-wtYAP*. The destination vector *pCAGGS-LoxP-Gap43-GFP-LoxP-IRES-nRFP* (Wong et al., 2015) was used as control plasmid and used to obtain the CA-YAP plasmid. To this end, it was digested by XhoI, and *pTOPO-mouse-wtYAP* was digested by SalI to obtain linearized wtYAP DNA. The opened destination vector (*pCAGGS–LoxP–Gap43-GFP–LoxP–IRES–nRFP*) and the linearized wtYAP DNA were purified by 2% agarose gel electrophoresis and ligated together to yield *pCAGGS–LoxP–Gap43-GFP–LoxP–wtYAP–IRES–nRFP*. This plasmid was then used to generate the CA-YAP-carrying plasmid (*pCAGGS–LoxP–Gap43-GFP–LoxP–CA-YAP–IRES–nRFP*) by replacing two serine residues, YAP-S112 and YAP-S382, with alanine residues, by PCR point mutagenesis.

To obtain the DN-YAP plasmid, *pcDNA3.1-pA83-dnYAP* (RBD12195) was obtained from the RIKEN BRC through the National Bio-Resource Project of the MEXT, Japan. In *pcDNA3.1-pA83-dnYAP,* YAP-S112 is replaced with alanine, and the transactivation domain of mouse YAP is replaced with the engrailed repression domain from *Drosophila*. *pcDNA3.1-pA83-dnYAP* was subcloned into the *pCR2.1-TOPO-TA* vector (Invitrogen) using PCR and adding BamHI sites to the 5′ and 3′ ends of dnYAP, creating an intermediate, non-expressing vector *pTOPO-mouse-DN-YAP*. The destination vector *pCAGGS-empty* (Niwa et al., 1991) was used as control plasmid and used to obtain the DN-YAP plasmid. To this end, it was digested by BglII, and *pTOPO-mouse-DN-YAP* was digested by BamHI to obtain linearized DN-YAP DNA. The opened destination vector (*pCAGGS-empty*) and the linearized DN-YAP DNA were purified by 2% agarose gel electrophoresis and ligated together to yield *pCAGGS-DN-YAP*.

#### HEK293T cell transfection

HEK293T cells were grown in DMEM (GIBCO) supplemented with 10% fetal calf serum and containing 1% penicillin/streptomycin (GIBCO 15140122) at 37°C in an atmosphere of 5% CO_2_ / 95% air. The transfection was performed with Lipofectamine 2000 reagent (Invitrogen). The day before transfection, cells were plated on a 24-well plate, 10^5^ cells per well, in the above cell culture medium. Cells were either lipofectamine transfected with CA-YAP only (600 ng per well) or co-transfected with CA-YAP and CAGGS-Cre (each 600 ng per well). After 48 h, cells were harvested and fixed in 4% PFA for 20 min. The cells were washed with 1x PBS and processed for further analysis within 24 h.

#### Tamoxifen preparation and administration

Tamoxifen powder, 200 mg, was dissolved in 10 mL of corn oil (Sigma, T-5648) under constant stirring at ≈40°C. Tamoxifen was administered to trigger activation of Cre recombinase in *Tis21*::CreER^T2 ±^ mouse embryos. Pregnant mice received tamoxifen (2 mg, 0.1 ml) orally by gavage at E12.5, i.e., one day before IUE, and once on the day of IUE (E13.5).

#### In utero electroporation of mice

Tamoxifen-treated pregnant mice carrying E13.5 embryos were anesthetized using initially 5% isoflurane (Baxter, HDG9623), followed by 2%–3% isoflurane during the IUE procedure. Endotoxin-free plasmids (Control, *pCAGGS–LoxP–Gap43-GFP–LoxP–IRES–nRFP*; CA-YAP, *pCAGGS–LoxP–Gap43-GFP–LoxP–CA-YAP–IRES–nRFP*) were mixed on the day of surgery with Fast Green (Sigma, 0.25% final concentration) to a final plasmid concentration of 2 μg/μl in 1x PBS. Using a borosilicate microcapillary (Sutter instruments, BF120-69-10) the DNA/Fast Green mixture was intraventricularly injected, which was followed by six 50-msec pulses of 30 V at 1 s intervals (BTX genetronics Inc., 45-0052INT), using a 3-mm diameter electrode (BTX genetronics Inc., 45-0487). After the IUE, the uterus was placed back into the abdominal cavity, and the peritoneum was sutured (VICRYL 5-0, V493H). Abdominal skin was closed with clips and animal received 100 μl of painkiller (Rimadyl 1 mg/ml). Pregnant mice were sacrificed by cervical dislocation at the indicated time points (E14.5-E17.5), and embryonic brains were dissected and fixed in 4% PFA, overnight at 4°C.

#### Verteporfin treatment of ferret and human neocortex in *ex vivo* free-floating tissue culture

An *ex vivo* free-floating tissue (FFT) culture system, adapted and modified from Long et al. (2018) and Schenk et al. (2009), was used to perform verteporfin treatment of embryonic ferret and fetal human neocortex tissue. E33 ferret brains were dissected, meninges removed, and the two hemispheres separated. Fetal human neocortex tissue of 11-13 wpc was cut into 2000-2500 μm-thick pieces (tangential dimension). Tissue was cultured in a whole-embryo culture incubator (Ikemoto RKI) in a rotating flask with 1.5 mL of SCM (for composition, see below), and incubated at 37°C in the presence of a humidified atmosphere consisting of 40% O_2_ / 5% CO_2_ / 55% N_2_, with continuous rotation at 26 rpm. Control flasks contained 10 μl of DMSO (Dimethyl sulfoxide, Sigma, 472301) added to the 1.5 mL of SCM. Verteporfin flasks contained 1 μM of verteporfin (Verteporfin, Sigma, SML0534) dissolved in 10 μl of DMSO, added to the 1.5 mL of SCM. FFT cultures were carried out for 48 h, with one change of SCM (containing either DMSO or DMSO plus verteporfin) after 24 h. After 48 h of FFT culture, tissue was fixed in 4% PFA overnight at 4°C.

The SCM medium used for FFT cultures contained: 84 mL of Neurobasal medium (GIBCO, 21103049) supplemented with either 10 mL of rat serum (ferret and human cultures) or 10 mL of 5x KnockOUT™ Serum Replacement (for human cultures, GIBCO, 10828028), 1 mL GlutaMAX™ (100x), 1 mL penicilin/streptomycin (100x) (GIBCO, 15140122), 1 mL N-2 (100x) (GIBCO, 17502048), 2 mL B-27 (50x) (GIBCO, 17504044) and 1 mL of 1 M HEPES-NaOH, pH 7.2, to yield a final volume of 100 ml.

#### In utero electroporation of ferrets

In utero electroporation of E33 ferret embryos was performed as originally established (Kawasaki et al., 2012, Kawasaki et al., 2013), with the modifications indicated below. Pregnant ferrets (with embryos at E33) were kept fasted for at least 3 h before the surgery and placed in the narcosis box with 4% isoflurane. Subsequently, they were positioned on the operation table and attached to the narcosis mask with 3% isoflurane and injected subcutaneously with analgesic (0.1 mL Metamizol, 50 mg/kg), antibiotic (0.13 mL Synulox, 20 mg/kg or 0.1 mL amoxicilin, 10 mg/kg) and glucose (10 mL 5% glucose solution). The ferret bellies were then shaved, sterilized with iodide and surgically opened. Then, the uterus was exposed. As the ferret uterus is pigmented, a transmitted light source was used for the visualization of embryos. Embryos were injected intraventricularly with a solution containing 0.1% Fast Green (Sigma) in sterile 1x PBS, 2 μg/μl of one of the endotoxin-free plasmids (CTRL-empty or DN-YAP) as indicated. To visualize electroporated cells, CTRL and DN-YAP plasmids were co-electroporated with CAGGS-EGFP (1 μg/μl), using a 5-mm diameter electrode (BTX genetronics Inc., 45-0489). Electroporations were performed with six 50-msec pulses of 100 V at 1 s intervals. Subsequently, the uterus was placed back in the peritoneal cavity, muscle layer with the peritoneum were sutured (VICRYL 4-0), after which the skin was sutured intracutaneously. Animals were carefully monitored until they woke up and then underwent postoperative care for the following 3 days. Pregnant females received subcutaneous injections of 15 mL of 5% glucose and the painkiller Metamizol (50 mg/kg, WDT, 99012) three times per day.

The embryos were obtained by cesarean section at E35, and the cerebral cortex was dissected and fixed in 4% PFA overnight at 4°C. To this end, the mother ferrets underwent a second surgery that followed the same pre-operative care, anesthesia and analgesia as the first surgery. The sutures from the first operation were removed and the uterus exposed, after which the embryos were removed by a caesarian section. Subsequently a complete hysterectomy was performed, after which the muscle layer with peritoneum and skin were sutured and the animal underwent the same post-operative care as after the first surgery. Animals were kept at the BMS of the MPI-CBG for at least two weeks after the second surgery after which they were donated for adoption.

#### Human tissue electroporation

*Ex vivo* electroporation of fetal human neocortical tissue (12-14 wpc) was performed as described previously (Kalebic et al., 2019). Briefly, neocortical tissue was placed in an electroporation chamber filled with sterile PBS, followed by addition to the apical side of the tissue of either the mixture of plasmids (pCAGGS-EGFP at 1.8 μg/μl together with the LacZ CRISPR/Cas9 plasmid (Kalebic et al., 2016) at 1.8 μg/μl) for control, or the YAP CRISPR/Cas9 KO plasmid (Santa Cruz Biotechnology, sc-400040; encoding Cas9, three gRNAs, and EGFP; at 1.8 μg/μl), all in PBS containing 0.1% Fast Green (Sigma). Immediately thereafter, electroporations were performed (36 V, ten 50 msec pulses with 1 s intervals), with the cathode on the apical side and the anode on the basal side of the tissue. After electroporation, the tissue was washed in PBS. The electroporated tissue was incubated in FFT culture for 72 h, followed by fixation in 4% PFA and processing for immunostaining.

#### EdU labeling

To analyze cell-cycle re-entry, one pulse of EdU was administered by intraperitoneal injection of 0.1 mL of EdU (1 mg/ml) into pregnant mice carrying E14.5 embryos, 24 h before the animal was sacrificed. At this stage of mouse cortical neurogenesis, the average length of the sum of S-phase, G2 plus M-phase of *Tis21*-GFP–positive APs and BPs is ≈4 h and ≈5 h, respectively (Arai et al., 2011). Hence, the time period of 24 h between the EdU pulse and the analysis is sufficient for the incorporated EdU to become inherited by daughter cells. To determine whether or not an EdU-labeled daughter-cell (see below for EdU detection) derived from an RFP-expressing mother cell re-entered the cell-cycle, Ki67 immunofluorescence was performed as is described below. EdU detection was performed on cryosections. After the incubation with secondary antibodies (see below), the tissue was fixed again, for 20 min with 4% PFA. EdU detection was carried out following the protocol of the Click-iT EdU kit with Alexa Fluor 647 (Invitrogen), as described previously (Arai et al., 2011).

#### Immunofluorescence on fixed cells and tissues

Transfected HEK293T cells were incubated with trypsin (GIBCO 25300054) (5 min), harvested, sedimented (300 x *g*), resuspended in 1x PBS, and fixed in 4% PFA for 10 min at room temperature. Cells were permeabilized with 0.3% Triton X-100 in 1x PBS for 10 min, followed by quenching in 0.1 M glycine in 1x PBS for 10 min. Primary antibodies were incubated for 2 h at room temperature, followed by incubation with secondary antibodies for 1 h, all in 1x PBS containing 0.2% gelatin, an additional 300 mM NaCl, and 0.3% Triton X-100 (PGNT buffer). Coverslips with the fixed, permeabilized and immunostained cells were washed with 1x PBS and mounted on glass slides using Mowiol.

Embryonic mouse and ferret brain and fetal human brain tissues fixed in 4% PFA were washed in 1x PBS, immersed in 30% sucrose, and kept overnight in sucrose at 4°C on a rocking platform. The sucrose-soaked tissue was embedded with Tissue-Tek solution (O.C.T., Sakura Finetek) and frozen at –20°C. Tissue was sectioned on a cryostat (20-μm cryosections). Frozen cryosections were rehydrated in 1x PBS. To be able to reliably detect nuclear epitopes, we routinely carried out an antigen retrieval protocol as follows. Cryosections were heated in 0.01 M Na-citrate pH 6.0 at 70°C for 45-60 min. After cooling to room temperature cryosections were permeabilized with 0.3% Triton X-100 in the 1x PBS for 30 min and quenched in 0.1 M glycine in 1x PBS for 30 min. Primary antibodies were incubated overnight at 4°C, followed by incubation with secondary antibodies for 2 h, all in PGNT buffer. After several washes in PGNT buffer and then in 1x PBS, cryosections were mounted on glass slides using Mowiol.

#### Total YAP versus phospho-YAP

For the comparison of total YAP versus phospho-YAP immunoreactivity and the determination of nuclear dephospho-YAP levels (Figure S2A and B), immunofluorescence was performed as follows. Cryosections of fixed mouse, ferret and human neocortex of the indicated developmental stages (1-2 cryosections per embryo/fetus) were incubated overnight at 4°C with two primary antibodies against YAP, a mouse monoclonal antibody recognizing total YAP (Abcam, ab56701, 1:100) and a rabbit polyclonal antibody recognizing only YAP phosphorylated at serine127 (phospho-YAP, Cell Signaling, 4911, 1:100), and a polyclonal goat antibody against Sox2 (R&D Systems, AF2018, 1:100), followed by incubation with the respective appropriate secondary antibodies, anti-mouse-Alexa Fluor 488, anti-rabbit Alexa Fluor 555 and anti-goat Alexa Fluor 647 (Molecular Probes), for 2 h at room temperature. After a series of washes in 1x PBS, sections were mounted on glass slides using Mowiol.

For the quantification of YAP immunoreactivity, one image per cryosection was taken and 30 randomly selected Sox2-positive nuclei in the SVZ were scored per cryosection. For both, the total YAP channel and the phospho-YAP channel, immunofluorescence background values were determined by averaging the fluorescence signals of 10 DAPI-stained nuclei in the CP per image (as CP nuclei lacked YAP immunoreactivity, see Figure 1); these background values were then subtracted from the respective total YAP and phospho-YAP immunofluorescence values obtained for each of the Sox2-positive nuclei per cryosection. To be able to relate the resulting, background-corrected, nuclear total YAP and nuclear phospho-YAP immunofluorescence values to each other, the mean immunofluorescence values for total YAP and for phospho-YAP from three representative areas of cytoplasm per image were determined. The ratio of mean cytoplasmic total YAP immunofluorescene value / mean cytoplasmic phospho-YAP immunofluorescene value was multiplied with the nuclear phospho-YAP immunofluorescence values to yield the adjusted immunofluorescence values for nuclear phospho-YAP. Then, for each nucleus, the adjusted immunofluorescence value for phospho-YAP was subtracted from the immunofluorescence value for total YAP, to yield the value for dephosphorylated, i.e., active, YAP.

#### Protein phosphatase treatment

To determine the proportion of YAP in Sox2+ nuclei in the SVZ that was in dephosphorylated form and hence active (Figure S2C), immunofluorescence was performed as follows. Cryosections (3 μm) of fixed mouse, ferret and human neocortex of the indicated developmental stages (1-2 cryosections per embryo/fetus) were subjected to antigen retrieval followed by permeabilization as described above. Cryosections were then treated with lambda protein phosphatase (New England Biolabs, P0753S) in a total reaction volume of 100 μl per cryosection, which consisted of 10 μl of 10x Protein MetalloPhosphatase buffer (50 mM HEPES pH 7.5, 10 mM NaCl, 2 mM DTT, 0.01% Brij 35; New England Biolabs, B0761S), 10 μl of 10 mM MnCl_2_, and either 10 μl of 400 units/μl of lambda protein phosphatase plus 70 μl of water (phosphatase treatment), or 80 μl of water (control treatment). To control for the specificity of phospho-YAP primary antibody (Cell Signaling, 13008), we incubated lambda protein phosphatase treated mouse sections with phospho-YAP primary antibody which was followed by secondary antibodies, anti-rabbit Alexa Fluor 488. This experiment showed absence of immunofluorescence signal in treated sections. For each neocortex sample per species, three cryosections were subjected to phosphatase treatment for 2 h at 37°C in a humidified chamber, and three other cryosections were subjected to control treatment. Cryosections were then washed in 0.3% Triton X-100 in 1x Tris-HCl buffered saline containing 0.2% gelatine (washing/blocking buffer), and blocked for 30 min in the same buffer followed by overnight incubation at 4°C with a mixture of two primary rabbit monoclonal antibodies diluted in washing/blocking buffer, one to detect total YAP (Cell Signaling, 14074, 1:100) and the other to detect phospho-YAP (Cell Signaling, 13008, 1:100), and polyclonal goat Sox2 antibody (R&D Systems, AF2018, 1:100), followed by incubation in washing/blocking buffer containing the respective appropriate secondary antibodies, anti-rabbit Alexa Fluor 488 and anti-goat Alexa Fluor 555 (Molecular Probes), for 2 h at room temperature. After a series of washes in 1x PBS, cryosections were mounted to glass slides using Mowiol. Protein phosphatase treatment of mouse E14.5 neocortex was found to completely abolish the immunofluorescence signal obtained with the phospho-YAP antibody, indicating of complete dephosphorylation of serine112.

For the quantification of the YAP immunoreactivity detected with the mixture of the total YAP plus phospho-YAP antibodies upon control versus phosphatase treatment, one image per cryosection was taken and 30 randomly selected Sox2-positive nuclei in the SVZ were scored per cryosection. For each neocortex sample per species, after determining the average value per cryosection, the mean of these average values for the three control cryosections was set to 100%, and the mean of the average values for the three phosphatase-treated cryosections was expressed relative to this. The reduction, upon protein phosphatase treatment, in the YAP immunofluorescence signal obtained with the sum of the two antibodies (total YAP plus phospho-YAP) indicates the contribution of phospho-YAP to this signal.

### Quantification and Statistical Analysis

#### Determination of germinal zones, apical and basal mitoses

Germinal zones were determined according to the differences in the cytoarchitecture as revealed by DAPI staining of nuclei. The VZ was defined as the zone of a pseudostratified epithelium where nuclei are elongated, densely packed and radially aligned. The SVZ was defined as the zone of non-radially aligned, rounded and less densely packed nuclei. In ferret and human, iSVZ and oSVZ were distinguished according to the density of nuclei, where the iSVZ comprises densely packed rounded nuclei and the oSVZ comprises sparse nuclei. The IZ was defined as the zone located between SVZ and CP, which had sparser nuclei than the SVZ (or the oSVZ in ferret and human). The CP was defined as the zone of densely packed rounded nuclei beneath the pial surface. Apical mitoses were defined as pVIM- or PH3-positive mitoses occurring within the VZ, and basal mitoses were defined as pVIM- or PH3-positive mitoses occurring within the SVZ (Figures S4F, S4G, 5B, 5D, 6C, 6E, and 6F). Delaminated GFP-positive cells were defined as GFP-positive cells lacking ventricular contact (Figure 7).

#### Quantifications

All quantifications were performed on 1-μm optical sections, with the exception of pVIM staining in Figures 6E and 6F where 5 optical sections were analyzed (5-μm Z stack). Quantifications were performed using the Fiji (Schindelin et al., 2012) software with the cell counter plugin and/or “measure” function.

For Figures 2D, 2G, 3D, 3H, 4F, 4G, S4D, S4C, and S4F, nuclei were quantified based on the expression of the respective marker combined with RFP expression. The scored double-positive nuclei (RFP plus selected marker) were expressed as a percentage of the total number of RFP-positive nuclei in the given germinal zone. Quantification was done on 200-μm wide images, oriented parallel to the apical surface.

Quantifications of RFP-positive nuclei in each zone (Figure S5D) were expressed as a percentage of the number of RFP-positive nuclei in the given zone over the total number RFP-positive nuclei in the cortical wall.

To determine if a nucleus is YAP-positive (Figure 1), we measured and averaged the YAP immunofluorescence intensity for 10 nuclei located in the CP as background. In the VZ or SVZ, we scored a nucleus as YAP-positive if the immunofluorescence intensity was at least two times higher than the background immunofluorescence. For quantification of YAP immunoreactivity, we acquired two-three images per embryo/fetus, and in each image (i.e., each cryosection) 30 randomly selected DAPI-positive (Figure 1D), Sox2-positive (Figure 1E), Tbr2-negative or -positive (Figures 1I–1K) nuclei in the VZ (Figures 1I and 1J) or in the SVZ (Figures 1D, 1E, and 1K) were scored. The values obtained were averaged for each embryo/fetus. Data are expressed as the percentage of 30 scored cells.

Data were tabulated in Excel (Microsoft, Redmond, WA) and analyzed in Prism 5 (GraphPad) software. Statistical analyses were performed using unpaired Student’s t test, Mann-Whitney *U*-test, and one-way ANOVA test.

## Supplemental Information

Document S1. Figures S1–S6 and Supplemental Methods

Document S2. Article plus Supplemental Information
