# Supplementary material for: YAP Activity Is Necessary and Sufficient for Basal Progenitor Abundance and Proliferation in the Developing Neocortex
Source: Cell Rep. 2019 Apr 23;27(4):1103–1118.e6. doi: 10.1016/j.celrep.2019.03.091 (PMC6486488; doi:10.1016/j.celrep.2019.03.091)
Supplement: Document S1. Figures S1–S6 and Supplemental Methods [file mmc1.pdf]

Cell Reports, Volume *27*

Supplemental Information

YAP Activity Is Necessary and Sufficient for Basal Progenitor Abundance and Proliferation in the Developing Neocortex

Milos Kostic, Judith T.M.L. Paridaen, Katherine R. Long, Nereo Kalebic, Barbara Langen, Nannette Grübling, Pauline Wimberger, Hiroshi Kawasaki, Takashi Namba, and Wieland B. Huttner

Supplemental Information

1) Supplemental figures S1 to S6 with legends
2) Methods S1

1) Supplemental Figures

Kostic et al. Figure S1.

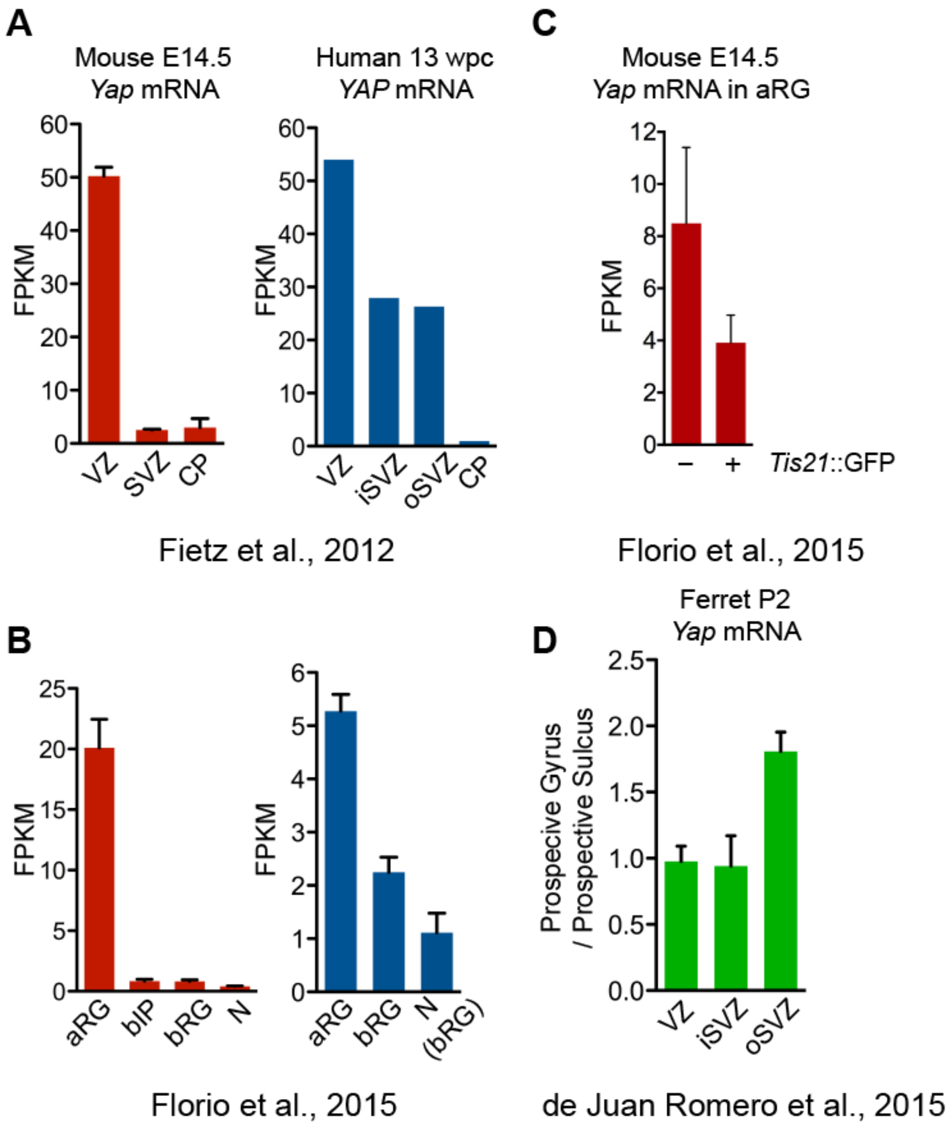

Figure S1. **Fetal human and postnatal ferret, but not embryonic mouse, neocortical BPs express *YAP* mRNA. Related to Figure 1.**

(A, B) FPKM values of *Yap*/*YAP* mRNA in the mouse E14.5 (left) and human 13 wpc (right) neocortical germinal zones (A, determined in Fietz et al. 2012) and cNPC subpopulations (B, determined in Florio et al. 2015); bIP, mouse cell fraction containing bIPs and other prominin-1 and DiI double-negative cell bodies; N, mouse neurons; N(bRG), human neuron fraction containing bRG in G1 (see Florio et al. 2015).

(C) FPKM values of *Yap* mRNA in mouse E14.5 *Tis21*::GFP-positive and -negative neocortical aRG (determined in Florio et al. 2015).

(D) Ratio of *Yap* mRNA levels in prospective gyrus / sulcus in ferret postnatal day 2 (P2) neocortex (determined in de Juan Romero et al. 2015).

Data are from one human transcriptome (A) or are the mean of 5 (A, C) and 4 (B) mouse transcriptomes, 4 (B) human transcriptomes, and 4 ferret transcriptomes (D); error bars indicate SD.

Kostic et al. Figure S2.

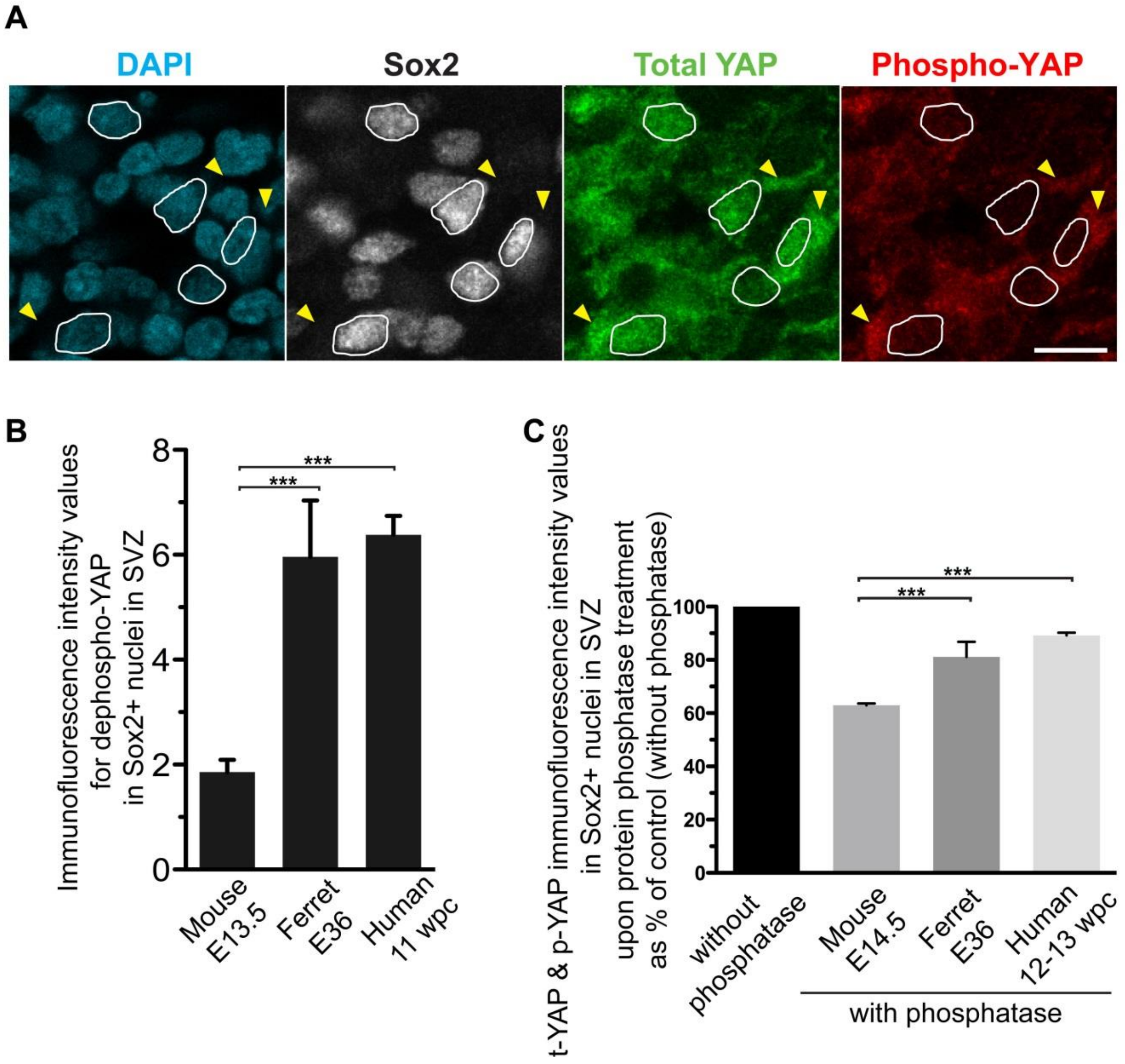

Figure S2. **A greater proportion of the nuclear YAP in BPs is dephosphorylated at serine127/112 in embryonic ferret and fetal human than embryonic mouse neocortex**. **Related to Figure 1.**

(A) Triple immunofluorescence for Sox2 (white), total YAP (i.e., YAP irrespective of phosphorylation; green) and phospho-YAP (P-serine 127; red), combined with DAPI staining (blue), on a cryosection of human 11 wpc neocortex; the SVZ is shown. Selected Sox2 and YAP (green) double-positive BP nuclei are outlined by white lines, as determined from DAPI-staining and/or Sox2 immunofluorescence. Yellow arrowheads indicate representative areas of cytoplasm used to standardize the immunofluorescence intensity for total YAP and phospho-YAP to each other. Note the barely detectable levels of phospho-YAP compared to total YAP immunoreactivity in the indicated Sox2-positive nuclei. Scale bar, 10 µm.

(B) Quantitation of dephospho-YAP in Sox2-positive BP nuclei in the SVZ of mouse E13.5, ferret E36 and human 11 wpc neocortex, by comparison of total YAP and phospho-YAP immunofluorescence. Cryosections were subjected to triple immunofluorescence for Sox2, total YAP and phospho-YAP as described in panel A for human neocortex. One to two images per embryo/fetus (1 image per cryosection) were taken, and 30 randomly picked Sox2-positive nuclei in the SVZ were scored per image, as follows. First, for each image, the mean immunofluorescence intensity values for total YAP and for phospho-YAP from three representative areas of cytoplasm (see arrowheads in A) were determined, to serve as internal standards for the comparison of nuclear total YAP and nuclear phospho-YAP. The ratio of these two values was used to adjust the immunofluorescence intensity values for nuclear phospho-YAP relative to the immunofluorescence intensity values for nuclear total YAP. Then, for each nucleus, the adjusted immunofluorescence intensity value for phospho-YAP was subtracted from the immunofluorescence intensity value for total YAP, to yield the value for dephospho-YAP. The values obtained for nuclear dephospho-YAP were averaged for each embryo/fetus. Data are the mean of 7 mouse, 4 ferret and 4 human embryos/fetuses. Error bars indicate SD; *** P <0.001 (one-way ANOVA test, post-hoc Tukey HSD).

(C) Quantitation of the proportion of phospho-YAP in Sox2-positive BP nuclei in the SVZ of mouse E14.5, ferret E36 and human 12-13 wpc neocortex, by determining the effect of protein phosphatase treatment on the total YAP plus phospho-YAP immunofluorescence signal. Cryosections were treated without (control) or with protein phosphatase, followed by double immunofluorescence for Sox2 and YAP. For YAP immunofluorescence, two rabbit monoclonal antibodies were used together, one recognizing YAP irrespective of serine127/112 phosphorylation (total YAP, t-YAP) and the other recognizing the serine127/112 phosphorylation site when phosphorylated (phospho-YAP, p-YAP). For each

neocortex sample per species, three cryosections without and three cryosections with protein phosphatase treatment were analyzed. For each cryosection, the immunofluorescence intensity values obtained with the sum of the two antibodies were measured in 30 randomly selected Sox2+ BP nuclei in the SVZ, and the average value per cryosection was determined. For each species, the mean of the three control cryosections is set to 100% (black column), and the mean of the three phosphatase-treated cryosections is expressed relative to this. Data are the mean of three neocortex samples per species; error bars indicate SD; ** $P < 0.01$, *** $P < 0.001$ (one-way ANOVA test, post-hoc Tukey HSD). Note that the reduction, upon protein phosphatase treatment, in the YAP immunofluorescence signal obtained with the sum of the two antibodies (total YAP plus phospho-YAP) indicates the contribution of phospho-YAP to this signal.

Kostic et al. Figure S3.

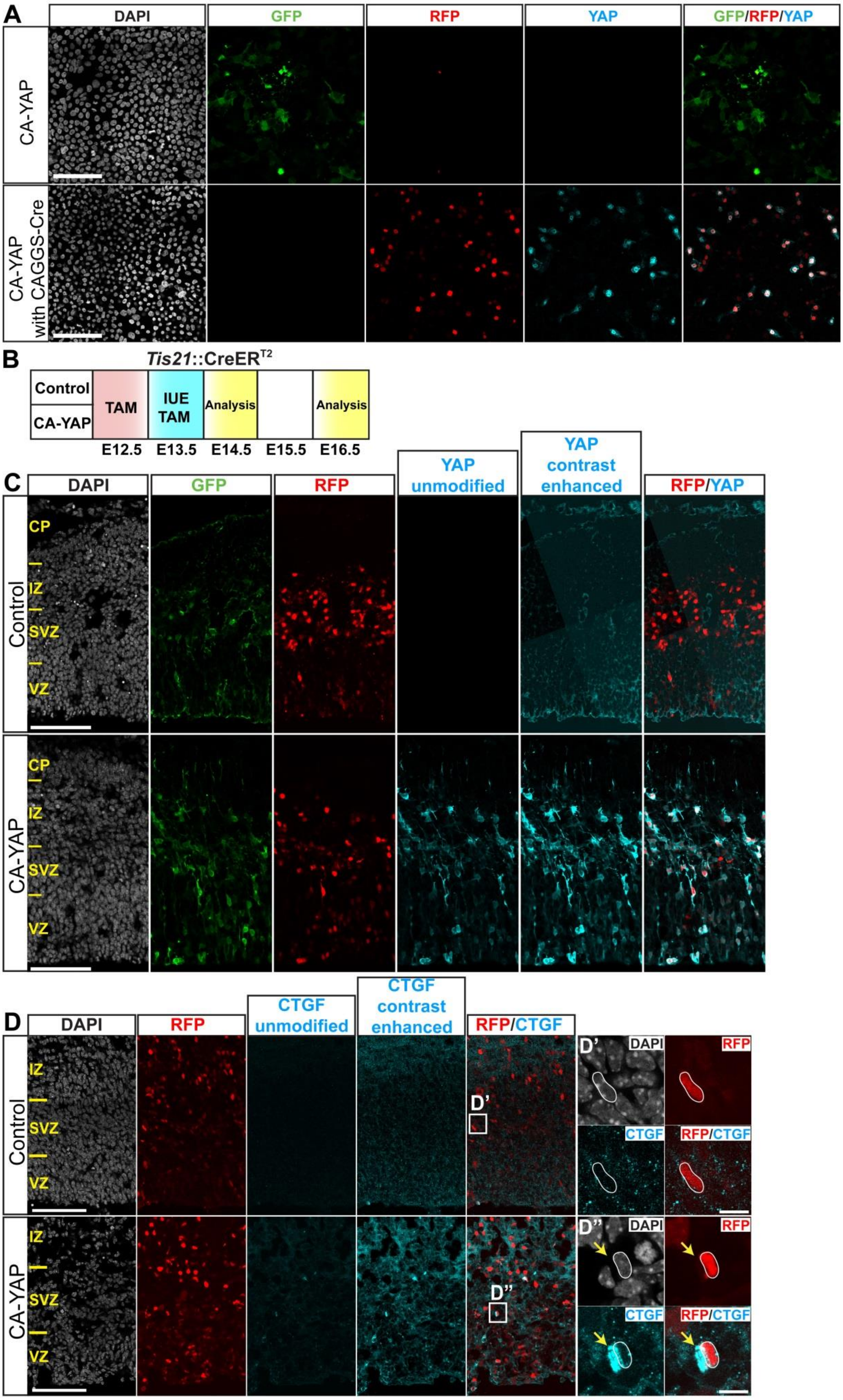

Figure S3. ***In vitro* and *in vivo* validation of conditional CA-YAP expression. Related to Figures 2, 3 and 4.**

(A) *In vitro* validation. HEK293T cells were transfected with the CA-YAP plasmid (see Fig. 2A) only (top row), or with the CA-YAP plasmid together with a CAGGS-Cre plasmid (bottom row), followed by triple immunofluorescence for GFP (green), RFP (red) and YAP (cyan), combined with DAPI staining (white), 48 h later. Whereas only membrane EGFP and no YAP expression was detected in CA-YAP–expressing plasmid alone (upper row; note that fluorescent laser intensity is too low to detect endogenous YAP protein expression), upon addition of CAGGS-Cre–expressing plasmid, YAP and RFP reporter expressions were visible, with mainly nuclear YAP immunofluorescence (lower row), suggesting an accurate recombination.

(B) Flow scheme of *in vivo* validation experiment. *Tis21*::CreER^{T2} heterozygous mouse embryos received tamoxifen (TAM) at E12.5 and E13.5, and the neocortex was subjected to IUE at E13.5 with control plasmid (C, D, top rows) or CA-YAP–expressing plasmid (C, D, bottom rows) (see Fig. 2A), followed by analysis at either E14.5 (C) or E16.5 (D).

(C) Triple immunofluorescence for GFP (green), RFP (red) and YAP (unmodified and contrast-enhanced images; cyan), combined with DAPI staining (white). In the control, expression of CA-YAP was not detected (upper row; note that in the unmodified image, fluorescent laser intensity is too low to detect endogenous YAP protein expression), whereas upon CA-YAP expression, YAP and RFP marker were mostly located in the VZ and SVZ (lower row). This data shows successful recombination of CA-YAP–expressing plasmid in vivo and efficient delivery of CA-YAP to BPs of mouse embryonic neocortex.

(D) Double immunofluorescence for RFP (red) and CTGF (unmodified and contrast-enhanced images; cyan), combined with DAPI staining (white). Boxes indicate areas in the SVZ that are shown at higher magnification in panels D' and D”; white lines outline an RFP-positive nucleus in the control (D’) or upon conditional CA-YAP expression (D”). After CA-YAP expression, we observed an increase in CTGF immunoreactivity in the cytoplasm and adjacent extracellular space in the SVZ (arrows) (D, D”), whereas in control CTGF exhibited no expression in the SVZ (D, D’), as expected because also mRNA of Ctgf was absent in the SVZ of embryonic mouse E14.5 neocortex (Florio et al., 2015). This suggests that CA-YAP promoted expression of CTGF in the embryonic mouse SVZ.

(A, C, D) Images are 1-µm optical sections. Scale bars, 100 µm in (A, C, D), 10 µm in (D’, D”).

Kostic et al. Figure S4

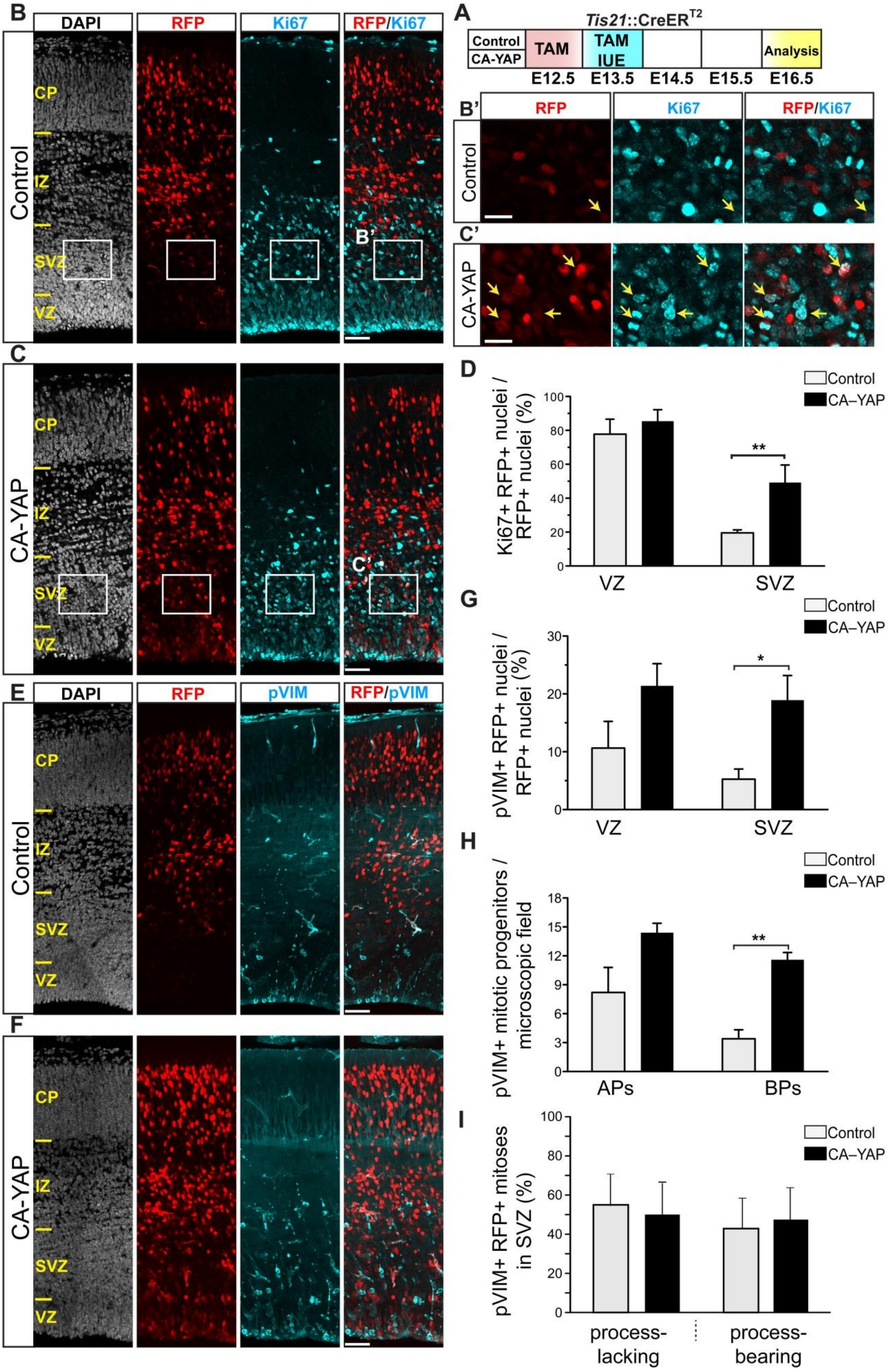

Figure S4. **Conditional CA-YAP expression in the BP-genic lineage of embryonic mouse neocortex promotes BP proliferation. Related to Figure 3.**

Tis21::CreER^{T2} heterozygous mouse embryos received tamoxifen at E12.5 and E13.5, and the neocortex was subjected to IUE at E13.5 with control plasmid (B, B', D, E, G, H) or CA-YAP–expressing plasmid (C, C' D, F, G, H) (see Fig. 2A), followed by analysis at E16.5, as shown in the flow scheme in (A).

(B, C, E, F) Double immunofluorescence for RFP (red) and either Ki67 (B, C) or pVIM (E, F) (cyan), combined with DAPI staining (white). Boxes in (B, C) indicate areas in the SVZ that are shown at higher magnification in panels B' and C'; arrows indicate selected RFP-positive nuclei that are Ki67-positive. Images are 1-µm optical sections. Scale bars, 50 µm in (B, C, E, F), 20 µm in (B', C').

(D, G) Quantification of the percentage of RFP-positive nuclei/cells that are Ki67-positive (D) and pVIM-positive (G) in the VZ and SVZ, upon control (light grey) and CA-YAP (black) electroporation. Two images (1-µm optical sections), each of 200 µm-wide field of cortical wall, per embryo were taken, and the percentage values obtained were averaged for each embryo.

(H) Quantification of the number of APs and BPs in mitosis, as revealed by pVIM immunofluorescence, per microscopic field (200 µm-wide field of cortical wall), upon control (light grey) and CA-YAP (black) electroporation. Two images (1-µm optical sections) per embryo were taken, and the values obtained were averaged for each embryo.

(I) Quantification of the percentage of pVIM- and RFP-positive cells in the SVZ upon control (light grey) and CA-YAP (black) electroporation (see panel G right for details of analysis) that either lack (left) or bear (right) a pVIM-positive cell processes.

Data are the mean of 5 embryos from five separate litters (D, G, H) or of 4 embryos from four sepatae litters (I). Error bars indicate SEM; * P <0.05, ** P <0.01 (Mann-Whitney *U*-test).

Kostic et al. Figure S5.

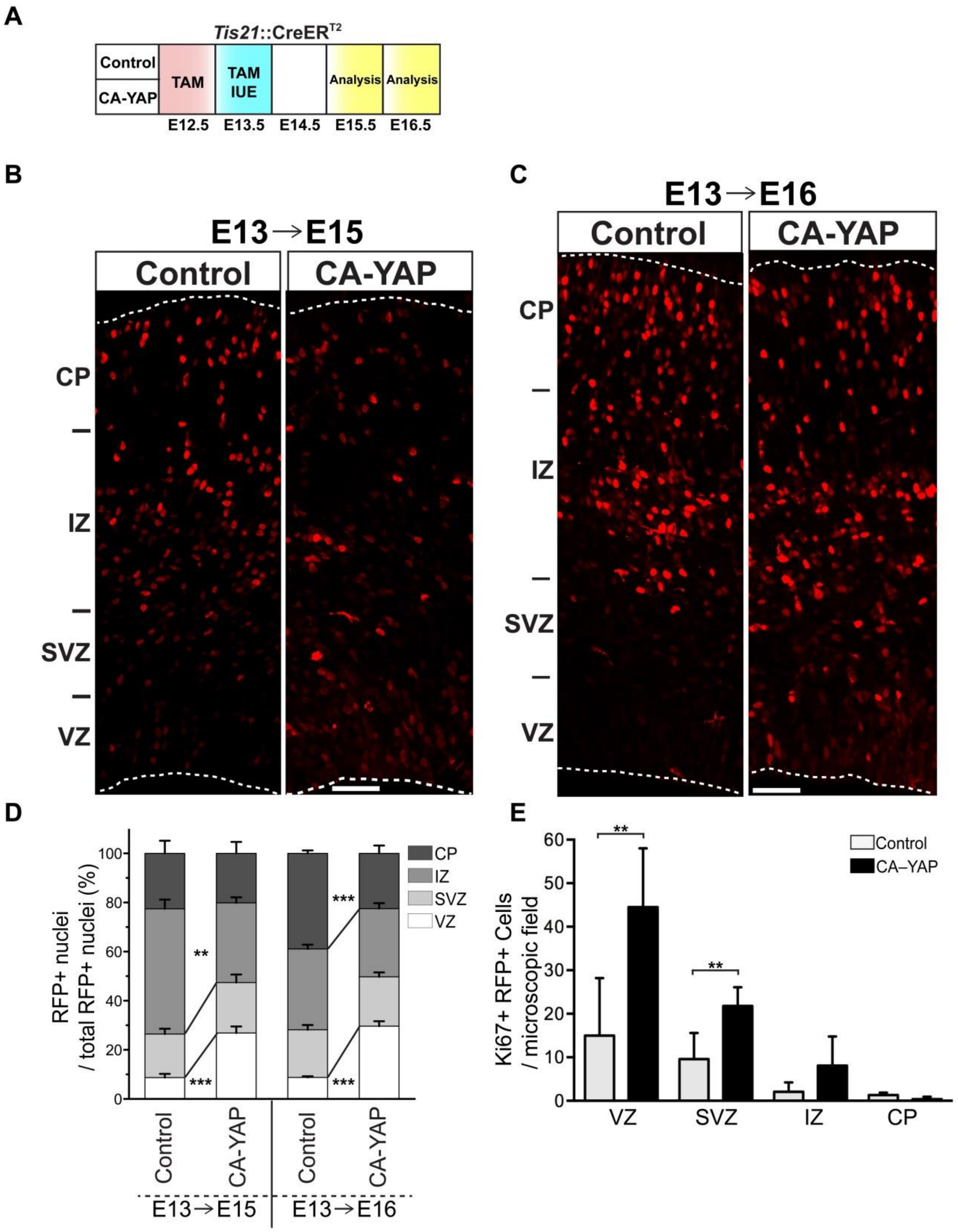

Figure S5. **Conditional CA-YAP expression in the BP-genic lineage of embryonic mouse neocortex increases the abundance of progeny in the VZ and SVZ. Related to Figures 2 and 3.**

(A) Flow scheme of experiments. *Tis21*::CreER^{T2} heterozygous mouse embryos received tamoxifen (TAM) at E12.5 and E13.5, and the neocortex was subjected to IUE at E13.5 with control plasmid (B, C, left) or CA-YAP–expressing plasmid (B, C, right) (see Fig. 2A), followed by analysis at either E15.5 (B, D left) or E16.5 (C, D right, E).

(B, C) Immunofluorescence for RFP (red) at either E15.5 (B) or E16.5 (C). Images are 1-µm optical sections. Scale bars, 50 µm.

(D) Quantification of the percentage of total RFP-positive nuclei in the cortical wall that are found in the VZ, SVZ, IZ and CP, either 2 days (left two columns) or 3 days (right two columns) after control or CA-YAP electroporation. Two images (1-µm optical sections) per embryo were taken, each of a 200 µm-wide field of cortical wall, and the percentage values obtained were averaged for each embryo. Data are the mean of 5 embryos from five separate litters.

(E) Quantification of the number of RFP-positive cells that are Ki67-positive in the VZ, SVZ, IZ and CP (200 µm-wide field of cortical wall), upon control (light grey) and CA-YAP (black) electroporation. Two images (1-µm optical sections) per embryo were taken, and the values obtained were averaged for each embryo. Data are the mean of 5 embryos from five separate litters.

Error bars indicate SEM; ** P <0.01, *** P <0.001 (unpaired Student's t-test).

Kostic et al. Figure S6.

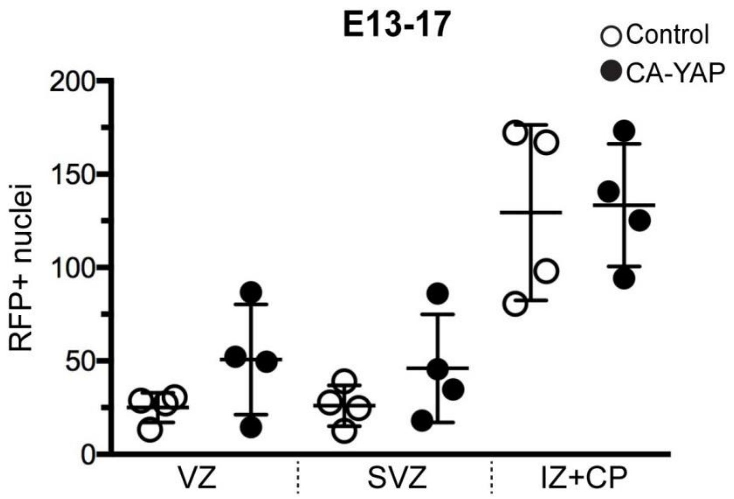

Figure S6. **Number of RFP+ nuclei, four days after IUE, in the embryonic mouse VZ, SVZ and IZ+CP. Related to Figure 4.**

Tis21::CreER^{T2} heterozygous mouse embryos received tamoxifen at E12.5 and E13.5, and the neocortex was subjected to IUE at E13.5 with control plasmid (open circles) or CA-YAP–expressing plasmid (solid circles) (see Fig. 2A), followed by analysis at E17.5. Two images (1-µm optical sections), each of a 200 µm-wide field of cortical wall, per embryo were taken, and the values obtained were averaged for each embryo. Data for 4 embryos from four separate litters, and the corresponding mean, are shown; error bars indicate SD.

Methods S1 Original and English-translated informed consent form

Patienteninformation und Einverständniserklärung

**Dies ist ein biomedizinisches Forschungsprojekt.
Nur Patienten, die sich freiwillig entschieden haben, daran teilzunehmen, werden einbezogen.
Bitte nehmen Sie sich Zeit, um Ihre Entscheidung zu treffen.**

1. Titel des Forschungsvorhabens

Entstehung von Nervenzellen während der Entwicklung des Gehirns (Kurztitel: NEUROGENESE).

2. Einladung zur Teilnahme an dem Projekt

Sie sind eingeladen, an diesem Projekt teilzunehmen, nachdem Sie vollständig hierüber informiert wurden. Mit Ihrem Einverständnis dafür, daß am Gewebe die unten beschriebenen wissenschaftlichen Untersuchungen durchgeführt werden dürfen, können Sie dazu beitragen, dass in der Zukunft eventuell eine bessere Diagnose und Therapie von Krankheiten, bei denen Nervengewebe zerstört wird (neurodegenerative Erkrankungen), möglich wird.

3. Einführung

- Während der Entwicklung des Gehirns entstehen u.a. Nervenzellen. Dies erfolgt beim Menschen auf andere Art und Weise als bei Labortieren wie z.B. der Maus. Deshalb sind die derzeitigen Informationen darüber, wie Nervenzellen im Gehirn der Maus entstehen, nur begrenzt auf den Menschen übertragbar.
- Ein Verständnis darüber, wie Nervenzellen während der Entwicklung des menschlichen Gehirns entstehen, ist aber eine Grundvoraussetzung dafür, in der Zukunft Nervenzellen zu regenerieren, die durch Erkrankungen wie z.B. Alzheimer, Parkinson oder Schlaganfall zerstört wurden. Ziel des Projekts ist es deshalb, Informationen über die Bildung von Nervenzellen aus den im frühen Embryonalstadium vorhandenen Vorläuferzellen zu erhalten.

4. Beschreibung des Forschungsprojektes

- Das Gewebe wird fixiert und feingeweblich daraufhin untersucht, welche Markermoleküle für die Bildung von Nervenzellen in welchen Vorläuferzellen vorkommen.
- Das Gewebe soll in Einzelfällen auch unfixiert auf Zellteilungs- und Zellwanderungsvorgänge hin untersucht werden, wobei ggf. geeignete Farbstoffe und Substanzen zur Anwendung kommen, die die Bildung von Nervenzellen beeinflussen können. Anschließend wird das Nervengewebe wie oben fixiert und untersucht.
- Das Gewebe wird ausschließlich für diese Laboruntersuchungen in vitro verwendet.
- Die beschriebenen Untersuchungen werden nach allen gültigen Regeln und Gesetzen durchgeführt.

5. Voraussehbare Risiken und Beschwerden

- Durch Ihr Einverständnis dafür, daß am Gewebe die beschriebenen wissenschaftlichen Untersuchungen durchgeführt werden dürfen, gibt es für Sie keinerlei zusätzliche gesundheitliche Risiken oder Beschwerden.

6. Ziels des Forschungsprojektes

- Ziel des Projekts ist es, ein besseres Verständnis über die Bildung von Nervenzellen im menschlichen Gehirn zu erhalten. Dies ist auch eine Grundvoraussetzung dafür, in der Zukunft Nervenzellen zu regenerieren, die z.B. durch neurodegenerative Erkrankungen zerstört wurden.

7. Allgemeine Aspekte des Forschungprojektes

- Die Gewebeproben werden im Max-Planck-Institut für Molekulare Zellbiologie und Genetik, einer Partnerinstitution des Universitätsklinikums bzw. der Medizinischen Fakultät Carl Gustav Carus der TU Dresden, untersucht.
- Es werden alle anwendbaren Gesetze beachtet und der volle Datenschutz zugesichert. Die geplanten wissenschaftlichen Untersuchungen und ihre Ergebnisse werden keine Rückschlüsse auf Ihre Identität zulassen.
- Es ist möglich, dass die Ergebnisse der geplanten wissenschaftlichen Untersuchungen zu einem Erwerb von intellektuellen Eigentumsrechten (Patenten) und zur kommerziellen Nutzung von Ergebnissen führen könnten. Sie erhalten keine finanzielle Zuwendung.

8. Erwartete Vorteile

- Für Sie als Patientin gibt es keine Vorteile oder Einkünfte.
- Mit Ihrem Einverständnis für die beschriebenen Untersuchungen können Sie dazu beitragen, dass in der Zukunft eventuell eine bessere Diagnose und Therapie von bestimmten Nervenkrankheiten möglich wird.

9. Freiwillige Beteiligung

- Die Beteiligung an dem beschriebenen Forschungsprojekt ist völlig freiwillig. Eine Ablehnung hat keine nachteilige Wirkung auf Ihr Verhältnis mit dem Arzt oder dem Krankenhausteam oder auf Ihre Behandlung.
- Ein Rücktritt von der Teilnahme an dem beschriebenen Forschungsprojekt ist jederzeit ohne Angabe von Gründen möglich, ohne dass für Sie irgendein Nachteil entsteht.

10. Datenschutz

- Alle Daten zu den Gewebeproben (z.B. die Schwangerschaftswoche) werden vollständig anonymisiert. Den Wissenschaftlern, die die beschriebenen Untersuchungen durchführen, ist Ihre Identität gänzlich unbekannt. Eine Mitteilung über eventuelle Forschungsergebnisse an Sie persönlich oder an Verwandte ist nicht geplant.
- Eine Veröffentlichung eventueller Forschungsergebnisse erfolgt ohne Personenbezug oder andere Hinweise auf Ihre Identität. Alle geltenden Gesetze und Regelungen des Datenschutzes werden beachtet.

11. Versicherung

Es ist keine zusätzliche Versicherung im Zusammenhang mit dem Forschungsprojekt vorgesehen, da keine zusätzlichen, d.h. über die ohnehin vorgesehenen medizinischen Maßnahmen hinausgehenden, Risiken entstehen.

12. Ansprechpartner

Für jede Frage oder jedes Problem wird Ihr Arzt Ihnen selbstverständlich zur Verfügung stehen. Bitte wenden Sie sich an den folgenden Arzt:

Name des Arztes: ____________________

Klinik und Poliklinik für Frauenheilkunde und Geburtshilfe

Telefon: ____________________

Bitte nehmen Sie sich Zeit, um diese Informationen zu lesen, und zögern Sie nicht, bei Unklarheiten Ihrem Arzt weitere Fragen zu stellen. Sie sollten eine Kopie dieses Dokuments behalten, nachdem Sie und Ihr Arzt unterschrieben haben.

Einverständniserklärung

Teilnahmebestätigung am Forschungsprojekt NEUROGENESE

Ich bin vollständig über das Forschungsprojekt NEUROGENESE und die geplanten wissenschaftlichen Untersuchungen an dem von mir zur Verfügung gestellten Gewebe informiert worden. Ich erhielt ausreichend Zeit, meine Teilnahme zu überdenken. Alle meine Rechte sind mir eindeutig erklärt worden. **Meine Beteiligung ist völlig freiwillig und ich habe jederzeit die Möglichkeit, meine Zustimmung zurückzuziehen.** Dies hat keinen nachteiligen Einfluss auf die Beziehung zu meinem behandelnden Arzt.

Ich habe eine Kopie der Patienteninformation erhalten.

Ich bestätige meine Teilnahme am Forschungsprojekt NEUROGENESE. Ich wurde darüber aufgeklärt, daß mir durch meine Teilnahme keinerlei zusätzliche gesundheitliche Risiken oder medizinische Nachteile entstehen. Ich bin damit einverstanden, daß an dem von mir zur Verfügung gestellten Gewebe die mir erläuterten wissenschaftlichen Untersuchungen durchgeführt werden. Ich bin ferner damit einverstanden, daß eventuelle Forschungsergebnisse veröffentlicht werden, vorausgesetzt dies erfolgt ohne Personenbezug oder andere Hinweise auf meine Identität.

Ich weiß, dass ich keine finanziellen Zuwendungen erhalte.

Alle angesammelten Daten (persönlich, klinisch und über Gewebeproben) werden in meinem Interesse und gemäß der europäischen "Richtlinie zum Schutz natürlicher Personen bei der Verarbeitung personenbezogener Daten und zum freien Datenverkehr" und der nationalen anwendbaren Gesetze behandelt.

Meine Zustimmung entlässt den Projektträger nicht aus seiner Verantwortung, und ich behalte alle meine gesetzlich garantierten Rechte.

Ort, Datum: ____________________

Name des Patienten: ____________________ Name des Arztes: ____________________

Unterschrift: ____________________ Unterschrift: ____________________

Weitere Person, die am Aufklärungsgespräch teilnahm: ____________________ Unterschrift: ____________________

Patient information and consent

This is a biomedical research project. Only patients who have voluntarily decided to participate will be included. Please take the time to make your decision.

1. Title of the research project

Generation of neurons during brain development (short title: Neurogenesis)

2. Invitation to participate in the project

You are invited to participate in this project after you have been fully informed. By agreeing that the nervous tissue may be used for the scientific research described below, you can help that perhaps in the future a better diagnosis and therapy of diseases that destroy nervous tissue (neurodegenerative diseases) will become possible.

3. Introduction

During the development of the brain, neurons are being generated. This occurs in humans in a different manner than in laboratory animals such as the mouse. Therefore, the current knowledge about how neurons are being generated in the mouse brain has only limited relevance for humans.

However, understanding how neurons are generated during the development of the human brain is a prerequisite for regenerating, in the future, neurons which have been destroyed by diseases such as Alzheimer, Parkinson or stroke. The aim of the project therefore is to obtain information on the generation of neurons from the progenitor cells present in the early embryo.

4. Description of the research project

The tissue will be fixed and examined histologically to determine which marker molecules for the generation of neurons are expressed in which progenitor cells.

In individual cases, the tissue will also be examined unfixed to investigate cell division and cell migration processes, using suitable dyes and substances which may influence the generation of neurons. Subsequently, the nervous tissue will be fixed and examined as above.

The tissue will be used exclusively for these in vitro laboratory tests. The investigations thus described will be carried out in compliance with all legal rules and laws.

5. Foreseeable risks and discomfort

Due to your agreement that the tissue can be used for the described scientific investigations, no additional health risks or discomfort will arise.

6. Aims of the research project

The aim of the project is to gain a better understanding about the generation of neurons in the human brain. This is also a basic prerequisite for regenerating, in the future, neurons that have been destroyed, for example, by neurodegenerative diseases.

7. General aspects of the research project

The tissue samples will be examined at the Max Planck Institute of Molecular Cell Biology and Genetics, a partner institution of the University Hospital and the Faculty of Medicine Carl Gustav Carus of TU Dresden.

All applicable laws will be observed, and full data protection is assured. The planned scientific investigations and their results will not allow any inference as to your identity.

It is possible that the results of the planned scientific research could lead to the acquisition of intellectual property rights (patents) and the commercial exploitation of results. In this case, you would not receive any financial bonus.

8. Expected benefits

For you as a patient, there will be no benefits or income.

By agreeing to the research described above, you can contribute to making a better diagnosis and treatment of certain neurological disorders possible in the future.

9. Voluntary participation

Participation in the described research project is completely voluntary. A refusal will not adversely affect your relationship with the physician or hospital team, or your treatment.

Resignation from participating in the described research project is possible at any time without giving reasons, without any disadvantage for you.

10. Data protection

All data concerning the tissue samples (for example, the pregnancy week) will be completely anonymized. The scientists doing the described research will be completely unaware of your identity. A communication about possible research results to you personally or to your relatives is not planned.

A publication of possible research results would take place without personal reference or other indications as to your identity. All applicable laws and regulations of data protection will be observed.

11. Insurance

No additional insurance is foreseen in connection with the research project, as there will be no additional risks, that is to say, beyond the already planned medical measures.

12. Contact person

Of course, for any question or problem, your physician will be available for you. Please contact the following physician:

Name of physician:___________________
Department of Obstetrics and Gynecology
Phone: ___________________

Please take your time to read this information, and do not hesitate to ask your physician if anything should be unclear. You should keep a copy of this document after you and your physician have signed it.

Patient information and consent

Consent form

Confirmation of participation in the research project Neurogenesis

I have been fully informed about the research project "Neurogenesis" and the planned scientific investigations using the tissue I will provide. I have had sufficient time to consider my participation. All my rights have been clearly explained to me. **My participation is completely voluntary, and I have the possibility to withdraw my consent at any time.** This would have no adverse effect on the relationship with my attending physician.

I have received a copy of the patient information.

I confirm my participation in the research project "Neurogenesis". I have been informed that through my participation no additional health risks or medical disadvantages will arise. I agree that the tissue I will provide can be subjected to the scientific research that has been explained to me. I further agree that research results can be published, provided that this is done without personal reference or other information as to my identity.

I know that I will not receive any financial benefits.

All accumulated data (personal, clinical and regarding tissue samples) will be treated in my interest and in accordance with the European "Directive on the Protection of Individuals with regard to the Processing of Personal Data and on the Free Movement of such Data" and with the national applicable laws.

My consent does not relieve the project-executing organization from its liability, and I retain all my rights guaranteed by law.

Place and date…………………………………………………………………………………………

Name of the patient……………………………………………………………………………………

Signature………………………………………………………………………………………………

Name of the physician…………………………………………………………………………………

Signature………………………………………………………………………………………………

Additional person who participated in the pre-operation discussion……………………………..

Signature………………………………………………………………………………………………
